# Advances in intelligent recognition and diagnosis of skin scar images: concepts, methods, challenges, and future trends

**DOI:** 10.3389/fmed.2025.1667087

**Published:** 2025-09-04

**Authors:** Fuhua Hu, Yuan Shao, Junjie Liu, Jialong Liu, Xiaolong Xiao, Kaibing Shi, Yangzong Zheng, Jianfeng Zhang, Xuelian Wang

**Affiliations:** ^1^Hangzhou Plastic Surgery Hospital (The Affiliated Hospital of the College of Mathematical Medicine, Zhejiang Normal University), Hangzhou, Zhejiang, China; ^2^College of Mathematical Medicine, Zhejiang Normal University, Jinhua, Zhejiang, China; ^3^School of Computer Science and Technology (School of Artificial Intelligence), Zhejiang Normal University, Jinhua, Zhejiang, China; ^4^School of Mathematical Sciences, Zhejiang Normal University, Jinhua, Zhejiang, China; ^5^Puyang Institute of Big Data and Artificial Intelligence, Puyang, Henan, China

**Keywords:** artificial intelligence in dermatology, computer vision for skin analysis, dataset, medical image process, large-scale foundation model

## Abstract

Skin scars, resulting from the natural healing cascade following cutaneous injury, impose enduring physiological and psychological burdens on patients. This review first summarizes the biological classification of scars, their formation mechanisms, and conventional clinical assessment techniques. We then introduce core concepts of artificial intelligence, contrasting traditional machine learning algorithms with modern deep learning architectures, and review publicly available dermatology datasets. Standardized quantitative evaluation metrics and benchmarking protocols are presented to enable fair comparisons across studies. In the Methods Review section, we employ a systematic literature search strategy. Traditional machine learning methods are classified into unsupervised and supervised approaches. We examine convolutional neural networks (CNNs) as an independent category. We also explore advanced algorithms, including multimodal fusion, attention mechanisms, and self-supervised and generative models. For each category, we outline the technical approach, emphasize performance benefits, and discuss inherent limitations. Throughout, we also highlight key challenges related to data scarcity, domain shifts, and privacy legislation, and propose recommendations to enhance robustness, generalizability, and clinical interpretability. By aligning current capabilities with unmet clinical needs, this review offers a coherent roadmap for future research and the translational deployment of intelligent scar diagnosis systems.

## 1 Introduction

**Figure 1:**
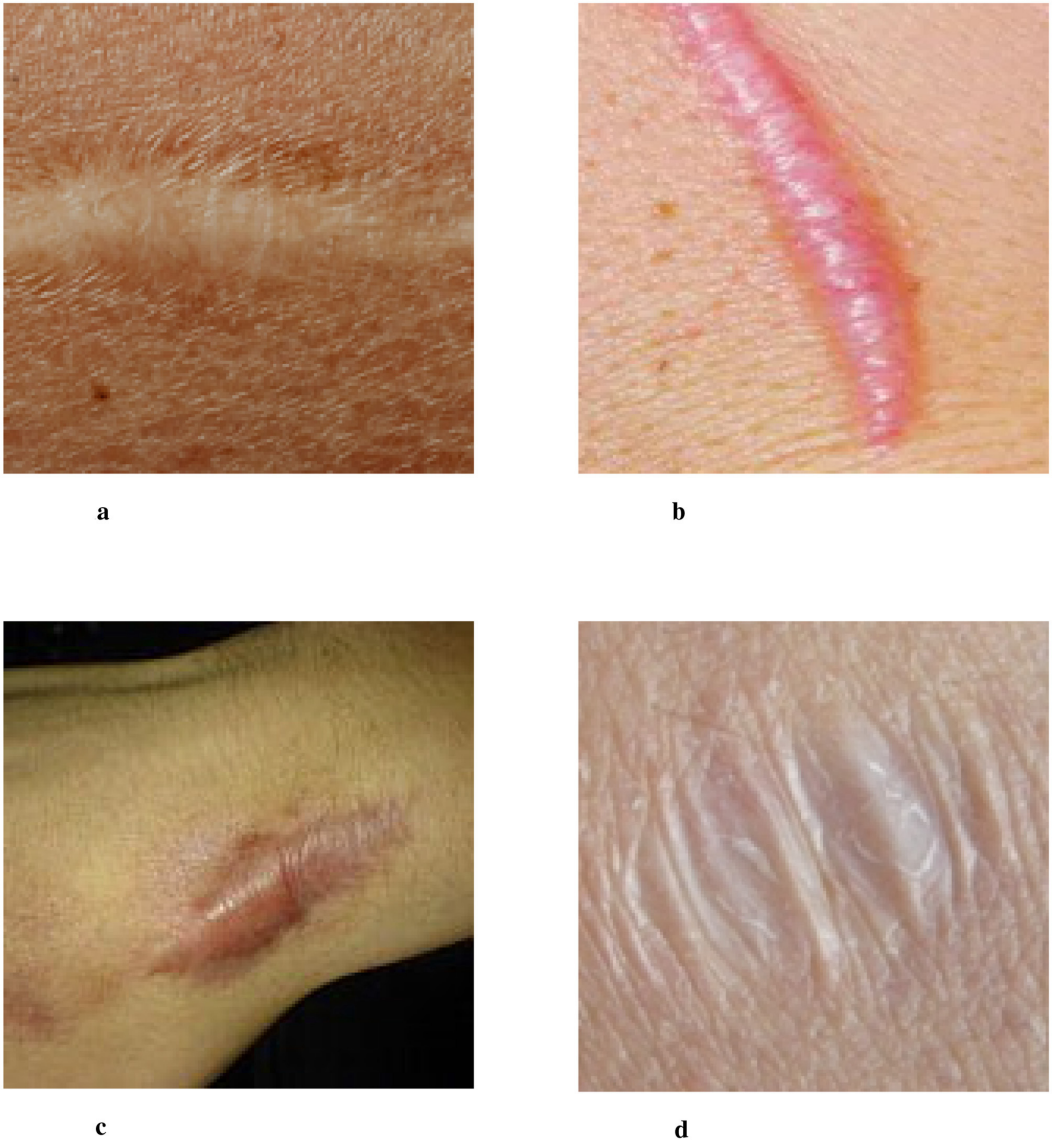
Four images of different scar types labeled a, b, c, and d. Image a shows a flat, pale scar. Image b depicts a raised, pinkish scar. Image c features a wide, darkened scar. Image d shows a wrinkled, white scar. Examples of clinical images of typical skin scars. The image data is sourced from publicly available datasets (Fitzpatrick 17k, etc.) and the Wikipedia entry “Scar.” **(a)** Normal Scar: Flat in appearance, with coloration closely resembling the surrounding skin, and a smooth surface texture. **(b)** Hypertrophic scar: characterized by a red or pink raised appearance that remains confined within the boundaries of the original wound. **(c)** Keloid: prominently elevated scar tissue that extends beyond the original wound margins, often darker in color. **(d)** Atrophic scar: marked by skin depression or indentation, commonly observed following the healing of acne or varicella (chickenpox) lesions.

Scarring is a natural part of the skin healing process after injury, where permanent fibrous tissue replaces damaged skin. This process occurs when the body produces either an excessive or insufficient amount of collagen during wound healing, resulting in visible marks or traces on the skin's surface (1). Scars represent the skin's attempt to restore structure and function by replacing damaged tissue. However, these scar tissues differ from normal skin in terms of structure and function, often manifesting as changes in color, texture, or elasticity. Scars can vary widely in type, depending on the underlying cause, and typically include normal scars, hypertrophic scars, keloids, and atrophic scars, among others (2–4). Several examples of clinical images typical skin scars are illustrated in Figure 1.

The impact of scarring extends beyond the skin's surface, profoundly affecting the psychological and emotional wellbeing of patients, particularly when scars are located on visible areas such as the face. Scarring can lead to self-esteem issues, social anxiety, and even depression (5–8). Furthermore, certain types of scars, such as keloids, may also cause physical discomfort, including pain or itching, which can significantly impair the quality of daily life (9).

Due to the complexity of scars and their profound impact on individuals, developing precise and objective scar assessment methods is of paramount importance. Traditional scar assessment relies on clinicians' experience and subjective judgment. Training a physician capable of accurately diagnosing dermatological conditions requires many years of education and clinical practice, involving exposure to thousands of patients (10). With advancements in artificial intelligence (AI) and deep learning technologies, intelligent recognition and diagnostic systems have emerged as a powerful tool in research and clinical practice, offering an efficient and standardized approach to scar assessment.

Intelligent diagnostic systems analyze skin images to automatically identify scar types and severity, providing clinicians with accurate and objective diagnostic information. This technology not only accelerates the diagnostic process but also improves accuracy and consistency, allowing more personalized and targeted treatment plans for patients. More importantly, intelligent recognition techniques offer a non-invasive and convenient evaluation method, significantly enhancing patient experience and satisfaction (11).

Although AI and machine learning have achieved remarkable improvements in medical image diagnosis, such as skin cancer detection and dermatological lesion analysis (12, 13), research on the intelligent recognition and diagnosis of skin scars remains relatively scarce. Our comprehensive review of the existing literature confirmed this gap. This research gap may be attributed to several factors:

1. Limited availability of high-quality datasets: Compared to other medical imaging domains, systematically collecting and annotating high-quality scar images poses significant challenges. Standardization, privacy concerns, and ethical considerations further complicate the process. Unlike imaging modalities such as computed tomography (CT) or magnetic resonance imaging (MRI), which follow strict acquisition protocols, scar images can be highly variable due to differences in lighting conditions, camera devices, angles, and distances. Additionally, since scars may appear in private or sensitive areas of the body, patient privacy concerns and ethical constraints pose barriers to dataset acquisition.
2. Disparity in clinical research priorities: Medical research resources are often allocated to conditions deemed more urgent or life-threatening. While scars can significantly affect a patient's quality of life, they may not always be prioritized as a critical medical issue, leading to relatively limited research efforts in this domain.

In addition to the scar-focused intelligent recognition methods reviewed herein, several representative studies in related domains have emerged. Li et al. (14) proposed a skin lesion classification model that combines multi-scale feature enhancement with an interaction Transformer module; Wang et al. (15) developed a segmentation network that fuses edge and region cues to improve lesion boundary delineation; Wang et al. (16) demonstrated a wide-field quantitative phase imaging approach using phase-manipulating Fresnel lenses to enhance tissue contrast; and Wu et al. (17) introduced a Dynamic Security Computing Framework based on zero-trust privacy-domain prevention and control theory to secure privacy data. Although these works do not directly target scars, their innovations in network architecture design, imaging modality enhancement, and system-level security offer valuable, transferable insights for the future development of intelligent scar analysis systems. We hope that this review will inspire further research and technological advancements, driving the application of intelligent medical technologies in scar diagnosis and management. By improving diagnostic accuracy and efficiency, these innovations have the potential to provide more effective, personalized, and patient-centered treatment solutions.

## 2 Classification, mechanism and traditional diagnosis of scars

### 2.1 Classification and formation mechanism

The formation of scars is a complex biomedical process that involves multiple stages of the skin's self-repair mechanism. Each stage is influenced by various factors, leading to different types of scars. This section introduces the formation mechanisms of various scar types, including normal scars, hypertrophic scars, keloids, and atrophic scars. Understanding these mechanisms is essential for leveraging AI and machine learning (ML) technologies to improve scar recognition and assessment, ultimately enabling more precise and personalized treatment strategies for patients.

Scars are generally classified into the following types:

1. Normal scars: these are the most common type of scars, typically resulting from minor cuts or incisions. Over time, they tend to fade and become less noticeable.
2. Hypertrophic scars (2): these scars form due to excessive collagen production during the healing process, resulting in thickened and raised tissue. However, unlike keloids, hypertrophic scars remain confined to the original wound boundaries.
3. Keloids (4): keloids are an overgrown form of scar tissue that extends beyond the original wound margins. They are typically firmer than normal skin and may be accompanied by pain or itching. Certain individuals are genetically predisposed to keloid formation, making them more susceptible to this condition.
4. Atrophic scars: characterized by a sunken appearance, atrophic scars form when the healing process leads to tissue loss. They are commonly seen as residual scars from chickenpox or acne (18, 19).

The process of scar formation follows the skin's natural wound healing mechanism, which occurs in several key phases (20, 21):

1. Inflammation phase: this phase begins immediately after an injury and lasts for several days. The affected area exhibits redness, swelling, heat, and pain as part of the inflammatory response. Immune cells, such as white blood cells and macrophages, infiltrate the wound site to remove dead cells, pathogens, and foreign debris. Additionally, inflammatory mediators release cytokines and growth factors that play a crucial role in stimulating subsequent cell proliferation and tissue formation.
2. Proliferation phase: during this phase, fibroblasts rapidly proliferate and synthesize extracellular matrix proteins, such as collagen, to establish a new tissue framework. Concurrently, new blood vessels form (a process known as angiogenesis) to supply nutrients and oxygen to the developing tissue. However, excessive fibroblast activity and collagen deposition can lead to the overgrowth of scar tissue, resulting in hypertrophic scars or keloids.
3. Remodeling phase: this final phase of wound healing can last from several months to years. Newly synthesized collagen undergoes structural rearrangement and maturation, making the scar tissue more closely resemble normal skin. Over time, scars may become flatter and softer, although in some cases, suboptimal healing can result in persistent depressions or protrusions.

**Figure 2:**
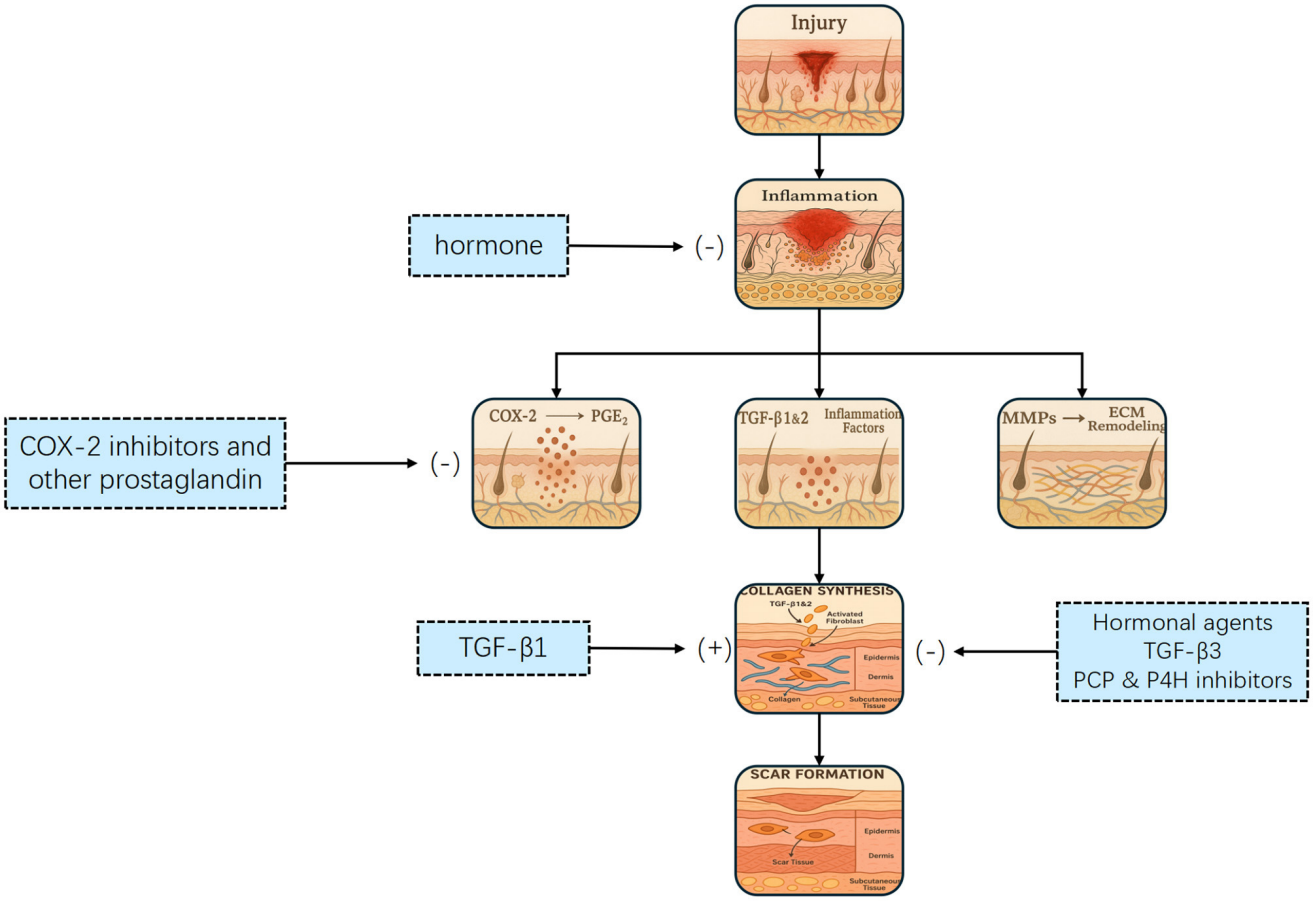
Flowchart illustrating the wound healing process, showing pathways from injury to scar formation. It includes inflammation, collagen synthesis, and ECM remodeling stages. Hormones, COX-2 inhibitors, and hormonal agents influence the process. The process of scar formation. Tissue injury initiates an inflammatory response that activates transforming growth factor-β (TGF–β) and other mediators, leading to fibroblast proliferation, migration, and differentiation. This promotes collagen synthesis and deposition, ultimately resulting in scar formation. Multiple therapeutic interventions targeting key steps—such as cyclooxygenase-2 (COX-2), TGF-β signaling, and fibroblast activity—may attenuate or prevent excessive scarring.

Scar formation is a dynamic and ongoing process, and extensive research has been conducted on the different stages of scar development, shown in Figure 2. A deeper understanding of these processes can significantly enhance the application of AI-driven technologies for intelligent scar recognition and diagnosis, ultimately leading to improved patient outcomes.

### 2.2 Traditional diagnostic methods

Before delving into intelligent scar recognition and diagnosis, it is essential to understand the foundation laid by traditional methods. Conventional scar assessment depends primarily on clinicians' experience and intuitive judgment, with visual and tactile examinations forming the core of the evaluation. Physicians first observe scar color, size, shape and contrast with surrounding skin to judge potential functional or aesthetic impact. Palpation then assesses hardness, texture, elasticity and temperature differences, helping to detect underlying inflammation or circulatory issues. Beyond these basic examinations, clinicians perform pain and sensory function tests and evaluate any functional limitations—for example, reduced joint range of motion due to perijoint scars. Standardized scales such as the Vancouver Scar Scale (VSS) and the Patient and Observer Scar Assessment Scale (POSAS) lend additional structure: the VSS scores vascularity, pigmentation, pliability and height, while the POSAS combines patient-reported symptoms with observer-rated scar characteristics (22, 23).

However, several key studies have quantified substantial inter-rater variability in these traditional scales. Draaijers et al. (23) evaluated 49 burn scar areas and reported single-observer reliability coefficients of *r* = 0.73 for the POSAS observer scale vs. *r* = 0.69 for the VSS (Cronbach's α = 0.69 and 0.49, respectively), indicating only moderate agreement among raters. Nedelec et al. (24) demonstrated that individual mVSS subscales yielded ICCs ≤ 0.30 and total mVSS scores ≤ 0.50, highlighting poor reproducibility of subjective metrics. More recently, Lee et al. (25) confirmed that both mVSS and POSAS fell below the acceptable Intraclass Correlation Coefficient (ICC) threshold of 0.70, whereas objective devices (e.g., ultrasound, colorimetry) achieved ICCs > 0.90.

These quantitative findings vividly illustrate the limitations of traditional visual and tactile assessment—namely, their reliance on subjective judgment and limited reproducibility. Consequently, there is a clear and growing need for AI-driven diagnostic approaches that can provide objective, consistent and fine-grained analysis of scar characteristics.

## 3 What is AI?

AI is a multidisciplinary field of computer science that aims to develop systems capable of performing tasks that typically require human intelligence. These tasks include reasoning, learning, problem-solving, perception, and language understanding. AI has evolved significantly over the past decades, driven by advances in computational power, data availability, and algorithmic innovations.

The field of AI encompasses several subdomains, including machine learning, natural language processing (NLP), computer vision (CV), expert systems, and robotics. Among these, machine learning—which enables systems to learn from data and improve their performance without being explicitly programmed—is one of the most transformative approaches, particularly in medical applications. With the development of deep learning, a subset of machine learning that utilizes neural networks to model complex patterns, AI has achieved remarkable breakthroughs in medical imaging, diagnosis, and personalized treatment (26).

**Figure 3:**
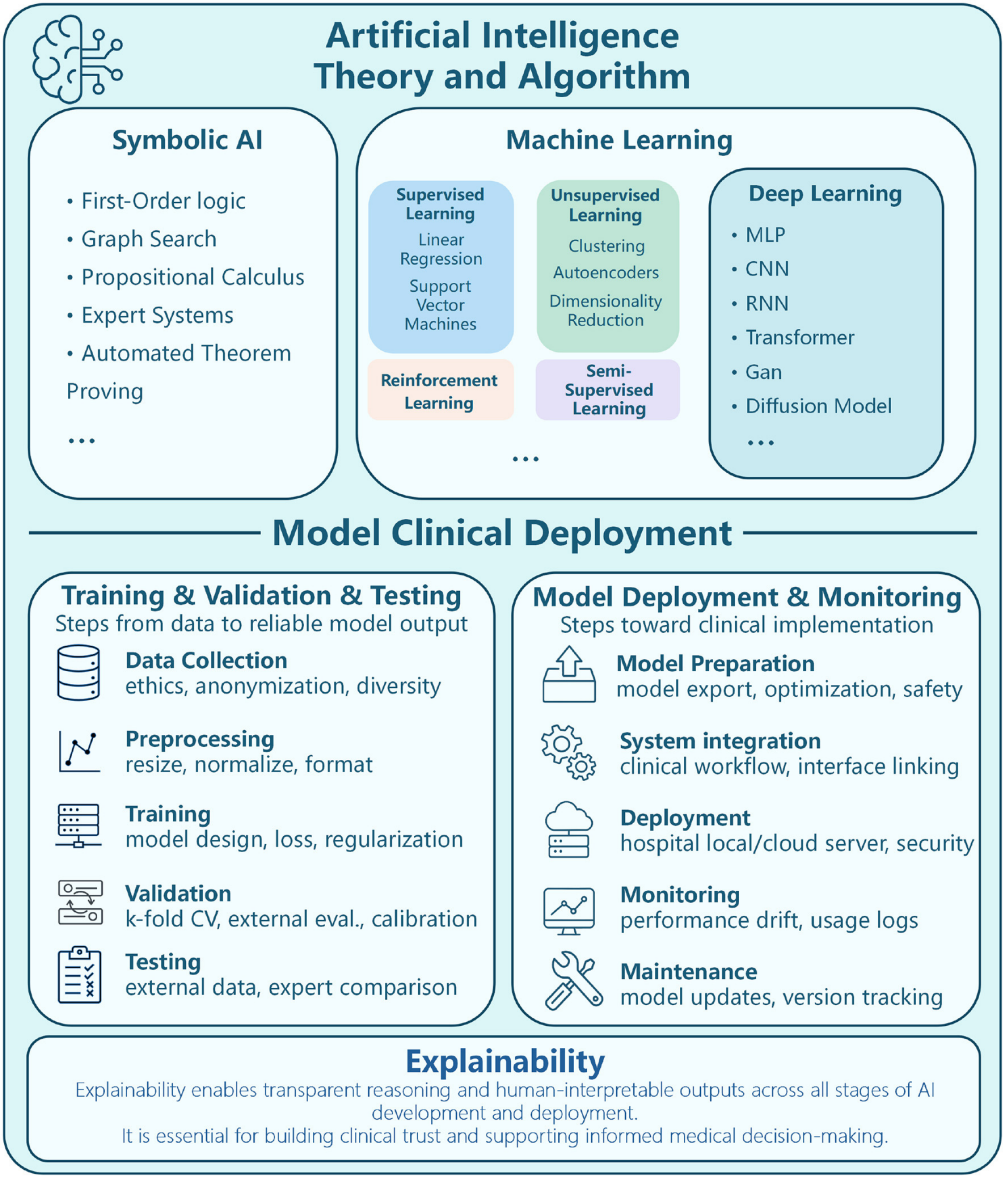
Diagram depicting AI theory, algorithm, and clinical deployment. Upper section outlines AI categories Symbolic AI (first-order logic, graph search), Machine Learning (supervised, unsupervised, reinforcement), and Deep Learning (MLP, CNN, RNN). Lower section details model clinical deployment, divided into Training/Validation/Testing (data collection, preprocessing, training, validation, testing) and Model Deployment/Monitoring (preparation, system integration, deployment, monitoring, maintenance). Emphasis on explainability for transparent reasoning and informed medical decision-making. Theoretical framework and methods of AI, ML algorithms, DL network framework.

As AI continues to advance, its integration into healthcare, including dermatology and scar assessment, holds great promise. The ability to automate medical image analysis and enhance diagnostic accuracy has positioned AI as a powerful tool in modern medicine, paving the way for more precise, efficient, and accessible healthcare solutions. The theoretical framework and methodologies of AI, as well as commonly used algorithms and network frameworks in ML and deep learning (DL), are illustrated in Figure 3.

### 3.1 Machine learning

Machine learning (ML) is a branch of artificial intelligence that enables systems to learn from data and improve their performance over time, without being explicitly programmed (27). ML models can be broadly categorized into three types based on how the data is used to train the model: supervised learning (28), unsupervised learning (29), and semi-supervised learning (30).

Supervised learning is the most common type of machine learning, where models are trained on labeled data, meaning each input data point has a corresponding output label. The goal is for the model to learn a mapping between inputs and outputs, so that it can predict the labels of new, unseen data. Common algorithms in supervised learning include linear regression (31), support vector machines (SVM) (32), k-nearest neighbors (KNN) (33), and decision trees (34). These algorithms are widely applied in tasks such as classification and regression, including applications like medical image classification (e.g., distinguishing benign from malignant tumors) and predicting patient outcomes (35). Supervised learning is essential when there is a large, labeled dataset available for training.

In contrast, unsupervised learning involves training models on data that does not have labeled outputs. The model's objective is to uncover the hidden structure or patterns within the data. Clustering and dimensionality reduction are typical examples of unsupervised learning tasks (36, 37). Algorithms such as k-means clustering (38), hierarchical clustering (39), and principal component analysis (PCA) are often used (40). In medical applications, unsupervised learning is helpful for segmenting medical images or identifying unknown patterns in complex datasets, such as detecting new disease subtypes based on genetic data (41).

Semi-supervised learning lies between supervised and unsupervised learning, where the model is trained on a combination of labeled and unlabeled data. This approach proves to be especially valuable when acquiring large labeled datasets is either difficult or costly, a situation that frequently arises in medical fields due to the scarcity of expert annotations. Semi-supervised learning can significantly improve the performance of the model by leveraging the abundance of unlabeled data. Techniques such as self-training and graph-based models are often employed in this approach. In healthcare, semi-supervised learning is increasingly used in medical image analysis, where only a small portion of the images may be annotated by experts, yet vast amounts of unannotated data are available (42).

### 3.2 Deep learning

Deep learning has emerged as a transformative advancement in artificial intelligence, enabling machines to perform complex tasks that traditionally required human expertise. As a subfield of machine learning, deep learning utilizes multi-layered neural networks to automatically extract hierarchical features from raw data, thereby obviating the need for manual feature selection. This ability to learn directly from data allows deep learning models to generalize across diverse applications. At its core, deep learning processes information through interconnected layers, with early layers capturing low-level features (e.g., edges, textures) and deeper layers identifying more complex patterns, such as object structures or diagnostic markers in medical data (43). This hierarchical representation learning allows deep learning to achieve superior performance across domains.

Deep learning has revolutionized fields like computer vision (44), natural language processing (45), and biomedical research (46). In healthcare, it has enhanced medical imaging, enabling automated disease detection, segmentation, and classification (47–49). It has also driven advancements in drug discovery, genomics, and personalized treatment strategies.

The rapid adoption of deep learning can be attributed to three main factors:

1. Powerful feature extraction: deep learning's capability to learn representations directly from raw data eliminates manual engineering, allowing models to capture complex patterns;
2. Growth in data and computational power: the surge in digital data and advancements in computational resources have fueled deep learning's success;
3. Continuous evolution of architectures: innovations in model architectures and training techniques, coupled with open-source frameworks, have accelerated the deployment of deep learning solutions.

As deep learning continues to evolve, ongoing research aims to improve model interpretability, reduce data dependency, and enhance architecture efficiency. Its continued integration into healthcare and other industries is paving the way for intelligent automation, improved decision-making, and new scientific breakthroughs.

**Figure 4:**
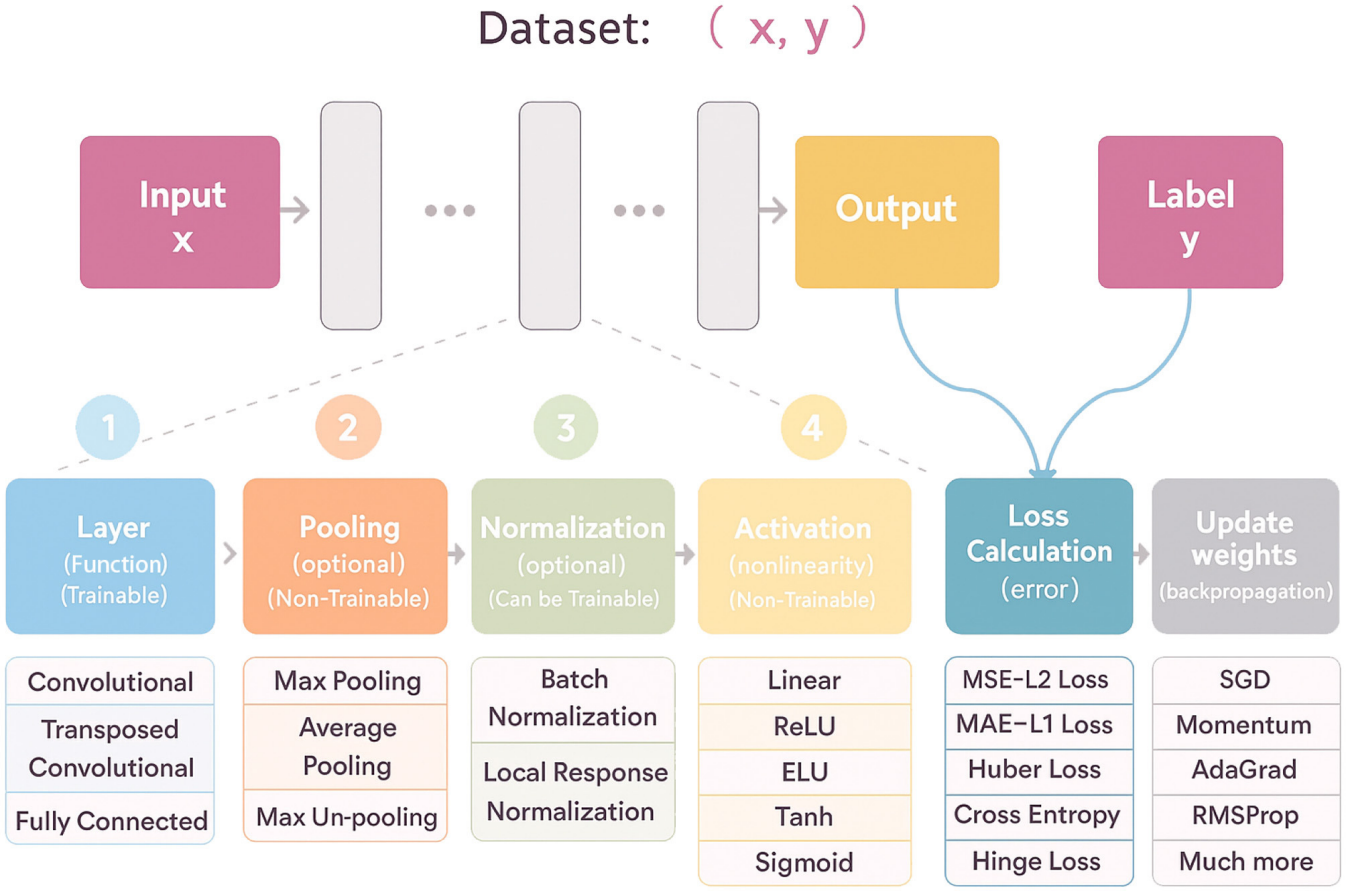
Flowchart illustrating a machine learning dataset process. Input x leads to an output and label y through stages: trainable layers with options like convolutional; optional non-trainable pooling; optional normalization; non-trainable activation functions; loss calculation; and weight updates via gradient descent methods. A visual overview of a typical deep learning workflow, illustrating the flow from input data through trainable and non-trainable components—such as convolutional layers, pooling, normalization, activation functions—to loss calculation and weight updates via backpropagation.

To illustrate the architectural diversity and historical evolution of deep learning, several representative models are summarized in Table 1, and a visual overview of a typical deep learning workflow shown in Figure 4.

**Table 1:** A few popular deep learning architectures.

**Network**	**Year**	**References**	**Description**	**Application in dermatology**
LeNet-5	1998	LeCun et al. (167)	Early CNN using convolution and pooling for image classification.	Used for dermatological image classification in early CNN studies (168).
AlexNet	2012	Krizhevsky et al. (169)	Introduced deep layers, ReLU, and GPU training for image classification.	AlexNet was used in skin disease classification, showing strong multi-class accuracy (170).
VAE	2013	Kingma (171)	Probabilistic encoder-decoder for data reconstruction and new sample generation.	Commonly used for generating synthetic dermatology images to augment training data (172).
GAN	2014	Goodfellow et al. (173)	Generates realistic data via adversarial generator-discriminator training.	Applied in dermatological image generation and integrated classification frameworks for multi-class skin disease detection (174, 175)
VGGNet	2014	Simonyan (176)	Deep CNN with stacked small filters for efficient feature learning.	Commonly used in dermatological image classification tasks due to its straightforward architecture and transferability (177, 178)
U-Net	2015	Ronneberger et al. (179)	Designed for biomedical image segmentation using an encoder-decoder with skip connections.	Widely used for skin lesion segmentation in dermatological imaging (180–182)
GoogLeNet	2015	Szegedy et al. (183)	Introduced the Inception module for multi-scale feature extraction with fewer parameters.	Used in skin image classification, often paired with lightweight models for better accuracy (184, 185)
Deep Q-Network	2015	Mnih et al. (186)	Combines Q-learning with deep networks to achieve human-level control in reinforcement learning.	Recently explored in dermatology for lesion segmentation, classification, and treatment recommendation tasks (90, 187, 188)
ResNet	2016	He et al. (189)	Introduced residual connections to train very deep networks, mitigating vanishing gradients.	Widely used in dermatology for classification and scar analysis due to its deep feature extraction capability (190–192)
DenseNet	2017	Huang et al. (193)	Uses dense connectivity for improved gradient flow and feature reuse.	Used for lesion classification and segmentation with efficient feature reuse (194, 195)
Transformer	2017	Vaswani (196)	Uses self-attention for efficient sequence modeling and spatial context learning.	Recently adopted in dermatology for capturing long-range dependencies in lesion segmentation and classification (123, 127)

## 4 Dataset

**Figure 5:**
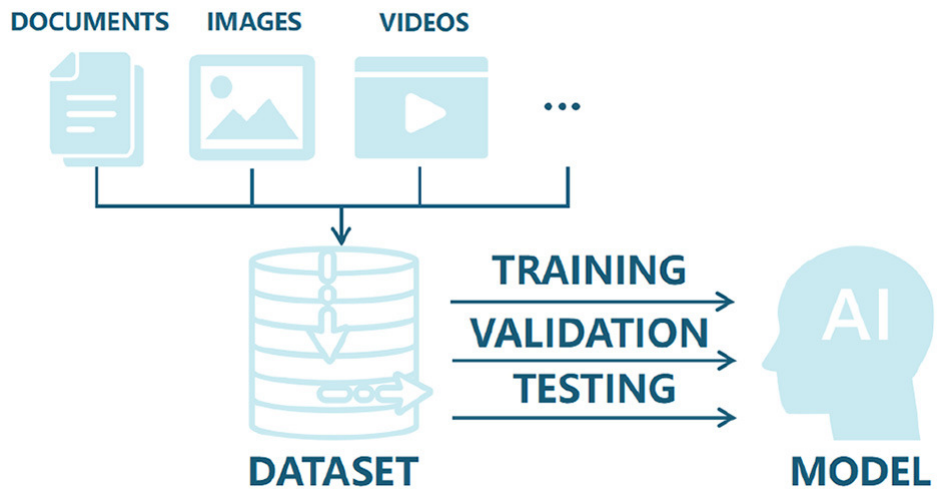
Diagram showing documents, images, and videos feeding into a dataset, which is used for training, validation, and testing an AI model. The process leads to a final AI model output. The foundational role of datasets in developing AI: from data collection to training, validation, and testing of intelligent systems.

Datasets are foundational to artificial intelligence, acting as carriers of information and knowledge that determine both the ceiling and the failure modes of downstream models (Figure 5). In current scar recognition research, however, most datasets are private and originate from hospital-affiliated projects with strict privacy and use restrictions. While such datasets may contain rich clinical detail, limited accessibility constrains reproducibility and the external validity of published findings.

Private datasets are typically collected and annotated by medical professionals. Their size and quality depend on patient volume, acquisition workflows, and the expertise of annotators. Although private collections may exhibit heterogeneous imaging conditions and granular labels, restricted access prevents independent validation and hampers community-wide progress.

**Figure 6:**
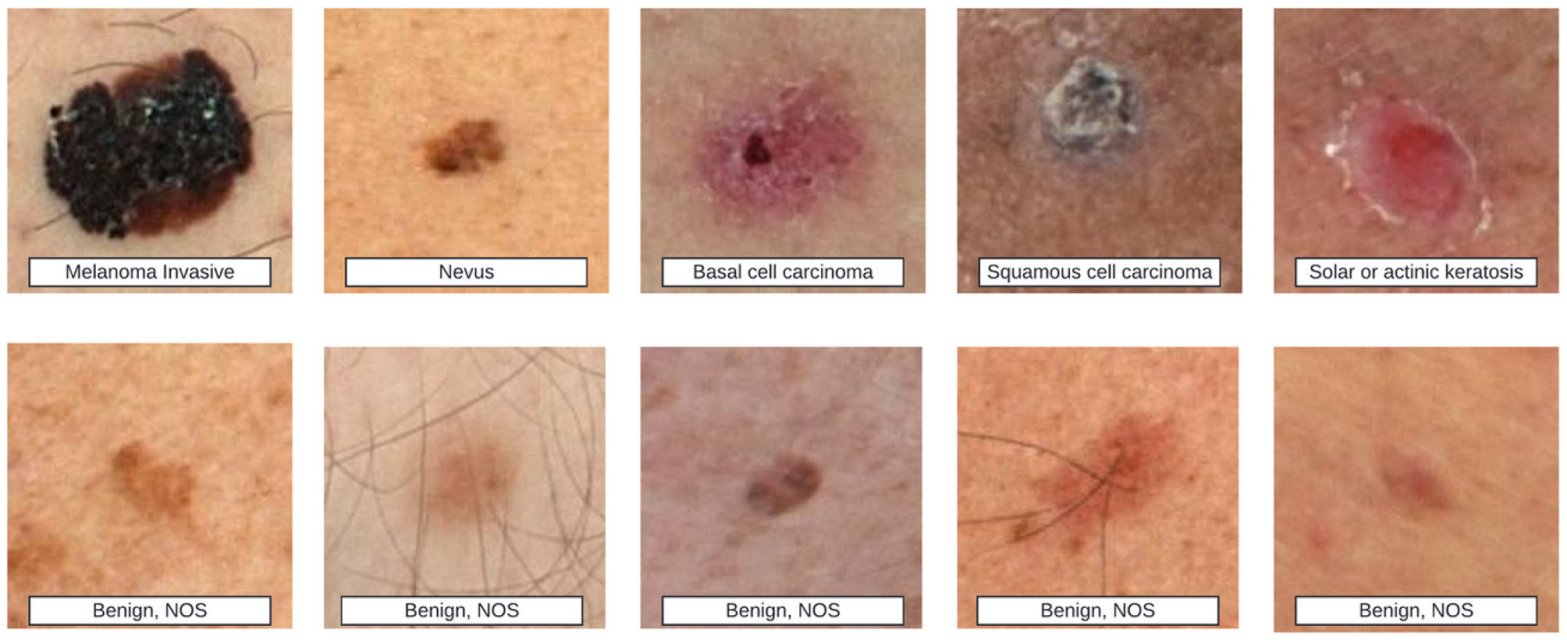
Top row shows various skin lesions labeled as melanoma invasive, nevus, basal cell carcinoma, squamous cell carcinoma, and solar or actinic keratosis. Bottom row contains different benign skin lesions labeled as benign, NOS. Example images from the ISIC dataset. The **top row** shows strong-labeled images, where detailed disease types are annotated. The **bottom row** shows weak-labeled images, where only benign or malignant status is provided. Some of these images may contain scar-like features, indicating their potential relevance to skin scar analysis.

Beyond scar-specific corpora, the broader dermatology field maintains several well-established public datasets (Table 2), some of which incidentally include scar images (see examples from the ISIC repository in Figure 6). These resources, however, were seldom curated with scars as a primary target, and often lack the metadata necessary to study fairness and generalization in scar analysis. Such metadata gaps extend beyond technical parameters and include clinically and technically salient variables-such as patient phenotype, scar architecture, and imaging conditions-whose omission can hinder comprehensive bias and generalization assessments.

**Table 2:** A few publicly available dermatology datasets.

**Dataset**	**Volume**	**Resolution**	**Scar images**	**Image types**	**Links**
ISIC	20,000+	Mixed	Not labeled	Dermoscopy	ISIC Archive
HAM10000 (197)	10,015	Mixed	Not labeled	Dermoscopy	Harvard
PH2	200	768 × 560	Not labeled	Dermoscopy	PH2 Database
BCN20000 (198)	18,946	Mixed	Not labeled	Dermoscopy	Nature
MED-NODE	170	Mixed	Not labeled	Dermoscopy	Kaggle
Dermofit Image Library	1,300	Mixed	Not labeled	Clinical	University of Edinburgh
SD-198 (199)	6,584	Mixed	Not labeled	Clinical	Papers with Code
PAD-UFES-20 (200)	2,298	Mixed	Not labeled	Clinical	ScienceDirect
fitzpatrick17k (201)	17,000	Mixed	123+	Clinical	Kaggle
DDI (202)	656	Mixed	About 50	Clinical	DDI

“*Not labeled"* indicates that the dataset may contain scar images, but does not provide explicit annotations for scars.

### 4.1 Representational diversity (skin tones)

A growing body of evidence shows that widely used dermatology datasets are skewed toward lighter skin tones (Fitzpatrick I–III), resulting in performance disparities on darker phenotypes (50). For instance, generative or discriminative models trained on imbalanced data can systematically underperform on Fitzpatrick IV–VI even when sample size is controlled (51, 52). To support fair evaluation in scar analysis, future datasets should (i) record skin phenotype explicitly (e.g., Fitzpatrick I–VI or validated proxies), (ii) target balanced sampling across tone strata, and (iii) require subgroup reporting (per-tone sensitivity/specificity, balanced accuracy, worst-group accuracy, and calibration).

### 4.2 Scar architecture coverage

Clinical scars are heterogeneous in type, etiology, maturity, and anatomical site. Representative types include hypertrophic, keloid, atrophic, and contracture scars. Common etiologies include surgical wounds, burns, and trauma. Using labels aligned with established clinical instruments such as POSAS and VSS (23, 53), and recording item-level attributes-thickness, vascularity, and pliability-improves both learning and interpretability. Dataset splits should be stratified by patient identity as well as by scar type and anatomical site to prevent shortcut learning, where background skin texture or body region inadvertently serves as a proxy.

### 4.3 Imaging settings and acquisition variability

Generalization in clinical use hinges on robustness to illumination and equipment variability. We recommend recording: device class (smartphone/DSLR/dermoscope), sensor and lens, optical setting (polarized vs. non-polarized, flash/ring light), resolution and compression, white-balance/exposure mode, use of color charts, scene context (rulers, dressings, tattoos, hair), and capture protocol. Such metadata enables (i) cross-device/lighting analyses, (ii) leave-one-device/site-out validation, and (iii) targeted data augmentation (color constancy, exposure jitter) evaluated against held-out domains rather than the training distribution (54, 55).

### 4.4 Multimodal and metadata-rich datasets (clinical photos, dermoscopy, and 3D)

Beyond routine photographic images, scar categorization benefits substantially from complementary modalities and structured metadata. Clinical photographs capture global color and texture together with contextual cues; dermoscopic images (polarized/non-polarized) reveal vascular and pigment structures that aid in distinguishing hypertrophic from keloid scars (56, 57). Three-dimensional surface imaging (e.g., stereophotogrammetry or laser profilometry) provides height and volume maps for objective quantification and treatment monitoring (58, 59). Cross-sectional modalities such as optical coherence tomography (OCT) and high-frequency ultrasound (HFUS, with elastography where available) capture subsurface morphology, thickness, and stiffness associated with activity and maturity (60–62). In parallel, aggregated meta-datasets in dermatology increasingly pair clinical and dermoscopic photographs or integrate multi-institution, multi-modality collections with standardized metadata, which improves skin-tone-stratified analyses and cross-site/device generalization (60, 63, 64). When paired with well-defined fields (anatomical site, etiology and maturity, Fitzpatrick or Monk Skin Tone, device/illumination/polarization, calibration targets), such resources provide stronger supervision for differentiating scar architectures, quantifying activity, and disentangling confounders due to lighting or device variability.

Despite these advantages, most existing scar datasets either lack the above modalities or do not release consistent metadata schemas, limiting fairness assessments across skin tones and external validity across clinics and equipment. We therefore advocate curating aggregated, multi-institution datasets that (i) include harmonized clinical photos, dermoscopy, and-where feasible-3D or cross-sectional imaging; (ii) adopt standardized acquisition protocols and per-image metadata fields (see Table 3); and (iii) support evaluation protocols that explicitly test cross-modality generalization, leave-one-site/device-out splits, worst-group performance (e.g., Fitzpatrick types IV-VI), and probability calibration. These recommendations align dataset design with downstream clinical reliability.

**Table 3:** Recommended metadata fields for scar-image datasets to support fairness assessment and generalization.

**Factor**	**Recommended metadata**	**Rationale**
Skin phenotype	Fitzpatrick type I–VI; color chart or reflectance proxy; optional self-reported skin phenotype	Supports subgroup analysis and balanced sampling across skin tones; enables reporting of fairness and probability calibration metrics
Scar architecture	Type: hypertrophic, keloid, atrophic, contracture; etiology: surgical, burn, trauma; maturity or stage; POSAS or VSS item scores: thickness, vascularity, pliability	Ensures coverage of clinically distinct phenotypes; increases label detail and interpretability
Anatomical site	Standardized body-region code or map	Controls differences due to body region; enables train, validation, and test splits that respect anatomical site
Acquisition	Device class and model; lens; resolution and compression; polarization; illumination or flash; white balance and exposure mode; presence of a color chart	Enables robustness analysis under device and lighting shifts; supports cross-device and cross-site validation-train on all other domains and test on the held-out domain
Context	Camera-to-lesion distance; background; presence of rulers, dressings, tattoos, hair	Mitigates shortcut learning, where models rely on background or scene cues rather than scar morphology
Annotations	Rater identifiers and training level; inter-rater agreement metrics; version of the annotation guideline	Documents label quality and reproducibility; provides traceable records for quality control and error analysis
Splits and governance	Patient-wise split; additional splits by site, device, and time; notes on consent and de-identification	Improves generalization to unseen clinical settings; ensures ethical and legal compliance; documents the origin and processing history of the data

Building on these considerations, future research should aim to develop standardized, multimodal, and metadata-rich meta-datasets, together with bias-aware evaluation frameworks.

## 5 Privacy constraints and ethical AI training

As noted above regarding dataset privacy and ethical approvals, contemporary medical artificial intelligence development must navigate stringent privacy regulations, e.g., general data protection regulation (GDPR), health insurance portability and accountability act (HIPAA), and ethical review processes, which restrict data sharing and centralization. To address these challenges, researchers have developed a range of privacy-preserving techniques, including federated learning, synthetic data augmentation, differential privacy, and encryption-based methods, each of which balances data utility, privacy guarantees, and computational overhead in its own way.

### 5.1 Federated learning for decentralized model training

Federated Learning (FL) enables multiple institutions to collaboratively train a global model by exchanging local model updates rather than raw patient data, thus minimizing privacy risks associated with central data aggregation (65). In medical imaging, FL frameworks have been successfully applied to histopathology and radiology datasets, maintaining performance comparable to centralized training while respecting data sovereignty (66, 67). Recent advances integrate transfer learning and adaptive aggregation to further improve accuracy across heterogeneous sites without compromising privacy (68).

### 5.2 Synthetic data augmentation

When real-world medical datasets are scarce or cannot be shared due to privacy constraints, synthetic data generated by Generative Adversarial Networks (GANs) can augment training sets. GAN-based augmentation has been shown to improve CNN performance in tasks such as liver lesion classification and chest X-ray analysis, boosting sensitivity and specificity on underrepresented classes (69, 70). Comprehensive reviews demonstrate that synthetic data not only increases data diversity but can also serve as an anonymization tool, enabling model training without exposing patient-identifiable images (71, 72).

### 5.3 Differential privacy and encryption techniques

Differential Privacy (DP) introduces carefully calibrated noise into model updates or outputs, providing quantifiable privacy guarantees against inference attacks. DP-enabled FL frameworks have demonstrated practical viability in complex medical image analysis, achieving performance on par with non-private methods while bounding privacy loss (66, 73). Encryption approaches, particularly Homomorphic Encryption (HE), allow computations to be performed directly on encrypted data, ensuring that raw data remain confidential throughout training and inference (74). Fully Homomorphic Encryption (FHE) schemes, though computationally intensive, have been successfully prototyped for optical coherence tomography(OCT) image classification and chest CT nodule detection, marking a step toward “zero-trust” AI in healthcare (75, 76).

### 5.4 Other emerging strategies

Beyond these core methods, secure multi-party computation (SMPC) and zero-knowledge proofs (ZKP) are gaining attention for enabling privacy-preserving analytics without revealing sensitive inputs (77). Concurrently, the development of synthetic cohort generation via diffusion models and advance in privacy-balanced data sharing agreements hold promise for ethically ground AI research while safeguarding patient rights.

By embedding these privacy-centric techniques into the AI lifecycle—from data augmentation to model deployment—researchers can better balance clinical innovation with ethical and regulatory imperatives, fostering trust and enabling broader adoption of AI in medicine.

## 6 Intelligent scar recognition and diagnosis

### 6.1 Search strategy

To ensure this review encompasses all relevant research on “Intelligent Recognition and Diagnosis of Skin Scars,” a multi-step search strategy was employed. Initially, a comprehensive search was conducted in databases such as Google Scholar, PubMed, Web of Science, and Science Direct. Keywords were systematically combined, including terms such as “skin scars,” “scarring,” “burn,” “wound,” “hypertrophic,” “keloids,” “atrophic,” “dermatology,” “intelligent,” “automatic,” “recognition,” “diagnosis,” “segmentation,” “detection,” and “image analysis.” Additionally, to broaden the search scope, auxiliary keywords like “computer vision,” “machine learning,” “deep learning,” and “artificial intelligence” were also included.

The search was limited to English-language publications from the past 5–10 years to ensure the inclusion of the most recent advancements in the field. The inclusion criteria for the selected papers were: (i) research on skin scars related to the detection, recognition, segmentation, and classification of prior damage, (ii) traditional image processing methods, (iii) conventional machine learning methods, (iv) deep learning methods, (v) digital image modalities, and (vi) articles published in well-defined, reputable journals.

**Figure 7:**
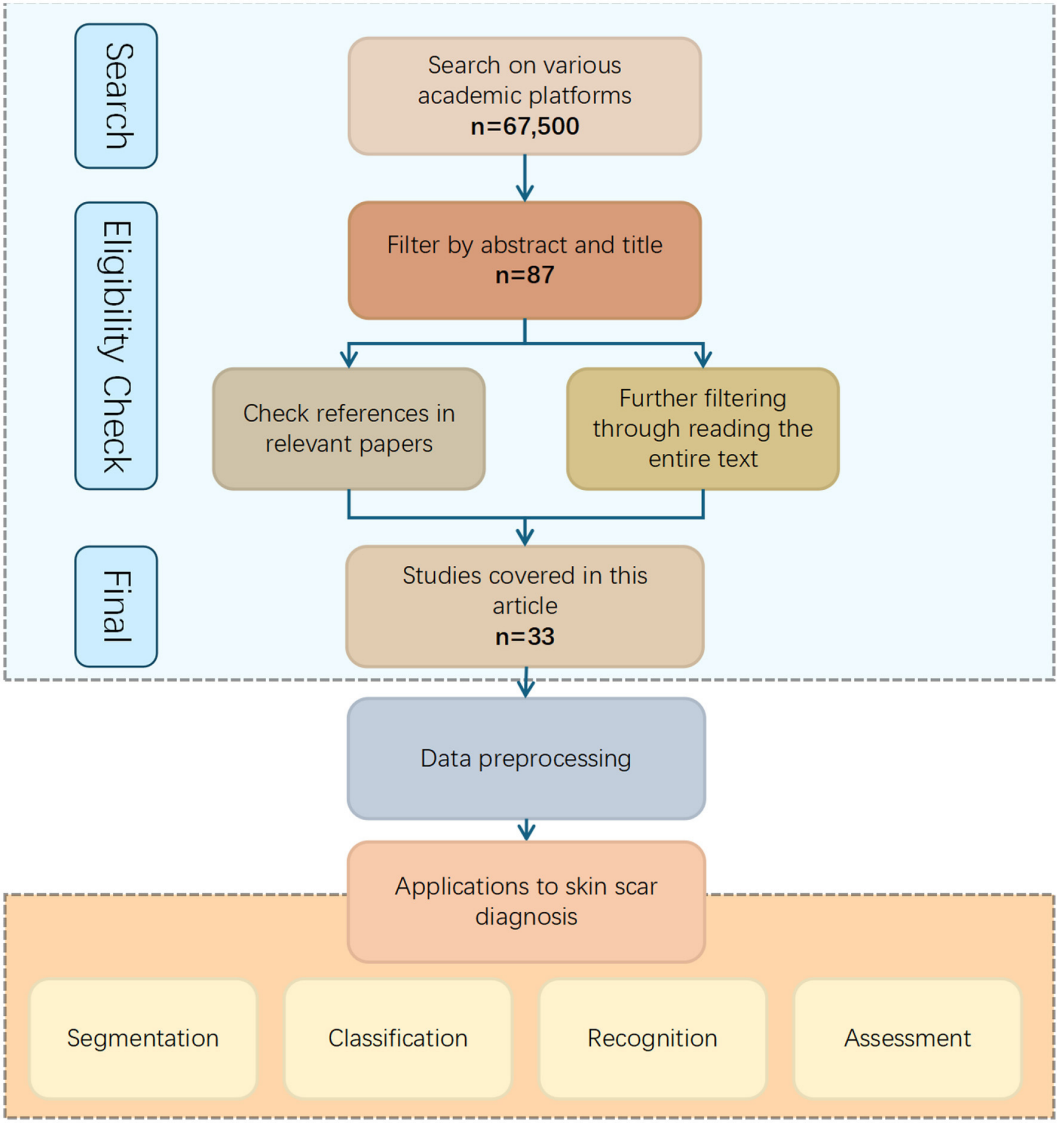
Flowchart depicting the process of selecting studies for skin scar diagnosis applications. Starting with a search on academic platforms yielding sixty-seven thousand five hundred results, filtering by abstract and title reduces it to eighty-seven, and further reference and text checks lead to thirty-three studies. These are used for data preprocessing, resulting in applications for segmentation, classification, recognition, and assessment of skin scars. Search strategy.

The initial search yielded 67,500 papers. The results were then refined through several rounds of screening: (1) removal of duplicate articles and inclusion based on the above criteria, (2) a thorough review of full-text papers to exclude studies with inadequate methodologies or irrelevant data, (3) manual examination of reference lists to ensure no relevant studies were overlooked. After these steps, a total of 33 articles were selected for inclusion. These articles comprehensively address all aspects of the topic, ranging from image acquisition and preprocessing to the application of traditional image processing and AI-based methods, as well as the evaluation of experimental results. The overall process is shown in the Figure 7.

This broad coverage of literature ensures the comprehensiveness and depth of this review, providing a solid foundation for future research directions.

### 6.2 Quantitative evaluation metrics for intelligent diagnosis

Quantitative evaluation metrics are essential tools used to objectively assess and compare the performance of AI-based diagnostic methods. The following summarizes the commonly used metrics in classification, segmentation, and regression tasks. To enhance clarity and compactness, the metrics are presented in Table 4, accompanied by unified symbol definitions.

**Table 4:** Summary of commonly used quantitative evaluation metrics in artificial intelligence.

**Task type**	**Metric**	**Definition**	**Formula**
Classification	Accuracy	Overall proportion of correct predictions	$\frac{TP+TN}{TP+TN+FP+FN}$
Classification	Sensitivity (recall)	True positive rate; important for avoiding missed diagnoses	$\frac{TP}{TP+FN}$
Classification	Specificity	True negative rate; reduces false positives	$\frac{TN}{TN+FP}$
Classification	Precision	Proportion of predicted positives that are true	$\frac{TP}{TP+FP}$
Classification	F1-Score	Harmonic mean of precision and recall; robust to class imbalance	$2\times \frac{\text{Precision}\times \text{Recall}}{\text{Precision}+\text{Recall}}$
Classification	AUC (ROC)	Area under the ROC curve; evaluates threshold-independent performance	–
Segmentation	Dice	Measures overlap between predicted and ground truth masks	$\frac{2|A\cap B|}{\left|A|+|B\right|}$
Segmentation	IoU	Ratio of intersection to union for segmentation regions	$\frac{\left|A\cap B\right|}{\left|A\cup B\right|}$
Regression	Mean Absolute Error (MAE)	Average of absolute differences between prediction and truth	$\frac{1}{n}\overset{n}{\underset{i=1}{\sum }}|y_{i}-{ŷ}_{i}|$
Regression	Root Mean Squared Error (RMSE)	Square root of the average squared differences; penalizes large errors more	$\sqrt{\frac{1}{n}\overset{n}{\underset{i=1}{\sum }}{\left(y_{i}-{ŷ}_{i}\right)}^{2}}$
Regression/ Measurement	Intraclass Correlation Coefficient (ICC)	Agreement between raters or repeated measures	Defined using mean square values; See below for full formula

To ensure clarity and consistency in interpreting the evaluation metrics presented above, the key symbols and variables used in the formulas are defined as follows:

- **TP** **(True positive)**: number of positive cases correctly predicted as positive.
- **TN** **(True negative)**: number of negative cases correctly predicted as negative.
- **FP** **(False positive)**: number of negative cases incorrectly predicted as positive.
- **FN** **(False negative)**: number of positive cases incorrectly predicted as negative.
- **A**, **B**: in segmentation tasks, *A* denotes the set of predicted pixels (or regions), and *B* denotes the ground truth set.
- ***y_i_***, **ŷ_*i*_**: The ground truth and predicted continuous values for the *i*-th sample, respectively.
- **n**: total number of samples or observations in the dataset.
- **k**: number of raters or measurement repetitions in reliability assessments.
- ***MS_R_***: mean square for rows (typically subjects) in the ICC calculation.
- ***MS_C_***: mean square for columns (typically raters) in the ICC calculation.
- ***MS_E_***: mean square error term in the ICC formulation, representing residual variance.

The *Intraclass Correlation Coefficient (ICC)* is a widely adopted statistical measure for assessing the reliability of quantitative measurements made by different raters or systems. In the context of intelligent diagnosis and clinical research, ICC is commonly used to evaluate either consistency or absolute agreement between observers. Various forms of ICC exist, depending on the statistical model employed, e.g., one-way vs. two-way analysis of variance(ANOVA), the nature of the raters (fixed vs. random effects), and whether the evaluation is based on single or average measurements.

Among these, **ICC(2,1)** is frequently utilized due to its suitability for assessing *absolute agreement* under a *two-way random-effects model*, in which both raters and subjects are assumed to be random samples. This variant is particularly appropriate in studies where generalization to a broader population of raters is desired. The corresponding formula is derived from an ANOVA decomposition that partitions the observed variance into components attributable to subjects, raters, and residual error. It is expressed as:


(1) $$\begin{array}{l} \text{ICC}\left(2,1\right)=\frac{MS_{R}-MS_{E}}{MS_{R}+\left(k-1\right)MS_{E}+\frac{k}{n}\left(MS_{C}-MS_{E}\right)} \end{array}$$

where *MS*_*R*_, *MS*_*C*_, and *MS*_*E*_ denote the mean square values for rows (subjects), columns (raters), and residuals, respectively.

In regression-based medical AI applications, **mean absolute error (MAE)** and **root mean squared error (RMSE)** are among the most frequently used evaluation metrics. MAE quantifies the average magnitude of prediction errors, offering direct interpretability in clinical units such as millimeters or severity grades. RMSE, due to its squared term, places greater emphasis on larger errors, making it more sensitive to outliers and thus useful in safety-critical predictions.

These metrics are particularly informative when assessing models that predict continuous clinical scores. For example, in scar severity prediction tasks, MAE values close to 1.0 and RMSE values around 1.4 may indicate that model outputs typically differ from expert-assigned scores by approximately one severity level, reflecting strong alignment with clinical judgment (78).

For classification tasks, the **receiver operating characteristic (ROC)** curve serves as a standard method to visualize model performance across varying decision thresholds. It plots the true positive rate (sensitivity) against the false positive rate (1− specificity), thus illustrating the trade-off between sensitivity and specificity.

The **area under the ROC curve (AUC)** condenses this information into a single scalar metric ranging from 0.5 (random performance) to 1.0 (perfect discrimination). AUC offers a threshold-independent assessment of a classifier's ability to distinguish between positive and negative cases. While the numerical value provides a summary, its interpretation is often more intuitive when supported by ROC visualizations.

**Figure 8:**
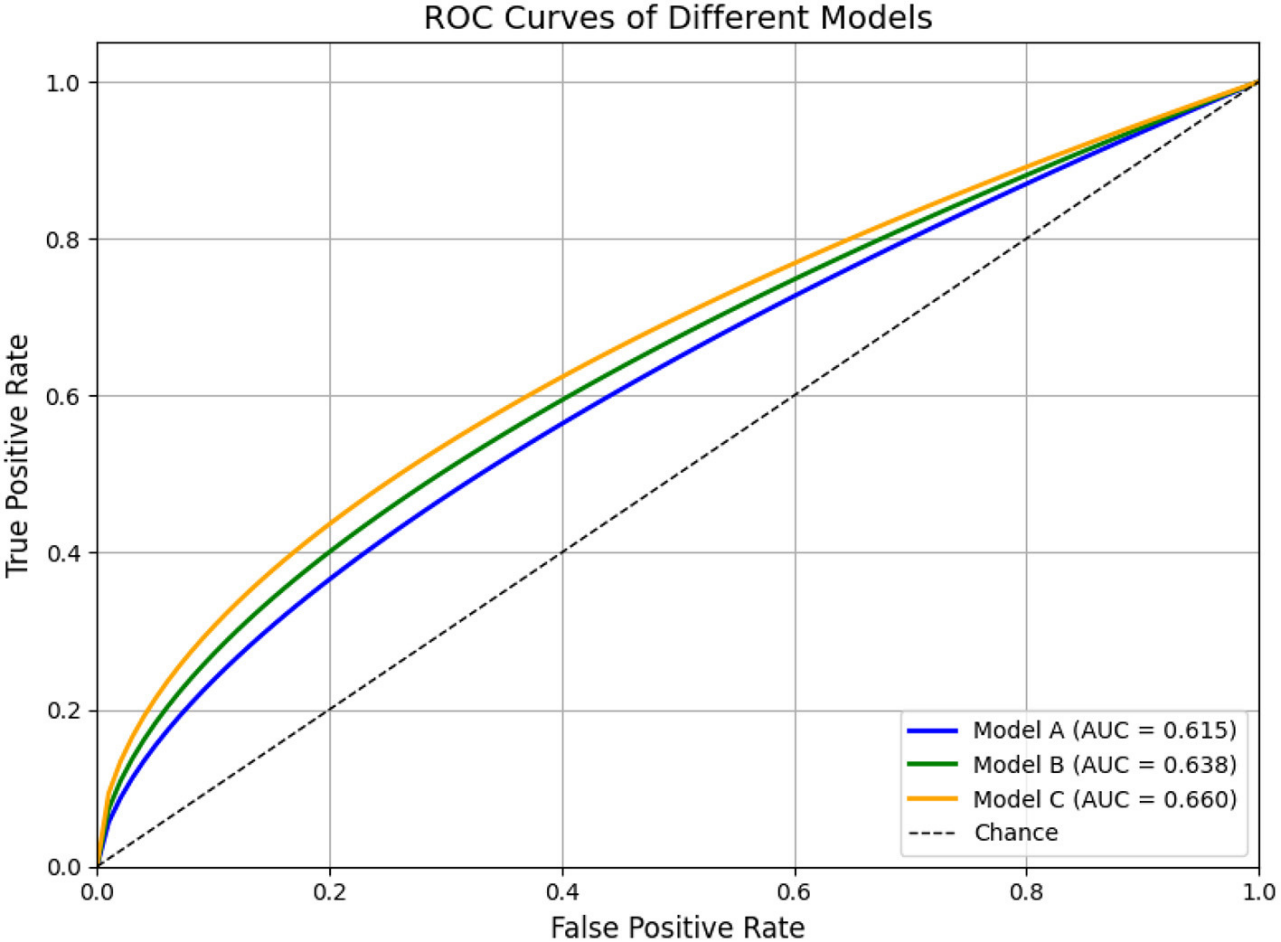
ROC curves for three models, showing the True Positive Rate versus False Positive Rate. Model A (blue) has an AUC of 0.615, Model B (green) 0.638, and Model C (orange) 0.660. A diagonal line represents chance. Simulated ROC curves of three hypothetical classification models (Model A, B, and C). Model A (blue) illustrates moderate classification performance (AUC = 0.80), Model B (green) shows improved overall discrimination (AUC = 0.88), and Model C (orange) demonstrates near-optimal performance (AUC = 0.97). The diagonal dashed line represents random classification (AUC = 0.5). This figure is intended for illustrative purposes to demonstrate how ROC curves and AUC values reflect the ability of models to distinguish between classes across various thresholds.

As illustrated in Figure 8, simulated ROC curves demonstrate how classifiers of varying quality (e.g., Model A, B, and C) differ in performance. Curves that approach the top-left corner correspond to higher AUCs and stronger discriminative power. Such visual representations are especially helpful when comparing models or assessing robustness across thresholds.

In segmentation tasks, the **Dice similarity coefficient** and **Intersection over Union (IoU)** are commonly employed to evaluate spatial overlap between predicted and ground truth regions. While related, these metrics serve different purposes and are not directly interchangeable.

Dice is particularly advantageous in scenarios with pronounced class imbalance—such as lesion or scar segmentation—where the target region occupies a small fraction of the image. It gives proportionally more weight to correctly identified positive pixels, making it sensitive to small structure detection.

In contrast, IoU is a stricter metric that penalizes both over- and under-segmentation. It is better suited for applications requiring precise boundary delineation, such as organ contouring or multi-class anatomical segmentation.

Therefore, metric selection should align with the clinical goal: **Dice** is more appropriate for detecting small or subtle targets, whereas **IoU** is preferred when spatial accuracy and structure completeness are prioritized.

These definitions provide a standardized interpretation of each metric, facilitating consistent comparison and critical evaluation of intelligent diagnostic systems across studies.

### 6.3 Unsupervised traditional machine learning methods

Unsupervised methods for scar segmentation and measurement typically exploit clustering or rule-based cues to delineate regions of interest without requiring annotated data. Ma et al. (79) presented a saliency-based segmentation framework for skin scars: Gaussian pyramid feature maps are clustered to produce saliency maps, which are then thresholded to isolate scar regions. Khan et al. (80) developed a segmentation pipeline based on fuzzy C-means clustering with an intelligent cluster-selection mechanism; they demonstrated that the Q (YIQ) and I3 (I1I2I3) chrominance components yield optimal cluster separation, achieving 92.63% segmentation accuracy on a set of 50 images. Chantharaphaichi et al. (81) proposed a rule-based image-processing scheme for acne lesion detection: grayscale and HSV(Hue, Saturation, Value) transformations are combined with brightness subtraction and size filtering to generate candidate lesion regions, which are then bounded with minimal operator intervention. Lastly, Jiang et al. (82) used unlabeled smartphone images of keloids to reconstruct three-dimensional models via parallel computing and extracted the maximum diameter, thickness, and volume. These measures showed excellent agreement with manual caliper and ultrasound assessments (ICCs > 0.95), indicating a highly repeatable, fully unsupervised measurement protocol.

### 6.4 Supervised traditional machine learning methods

Supervised approaches leverage hand-crafted feature extraction followed by classical classifiers trained on labeled examples. Liu et al. (83) combined local binary pattern (LBP) operators with wavelet-based texture analysis on multiphoton fluorescence microscopy images of scars; the resulting features were fed into a support vector machine (SVM) to distinguish scar tissue. Heflin et al. (84) introduced an automatic detection and classification system for scars, marks, and tattoos in unconstrained, forensic-style images by training classifiers on annotated samples from real-world scenarios. Abas et al. (85) fused entropy-based region-of-interest extraction with dual-tree wavelet frame (DWF) and gray-level cooccurrence matrix (GLCM) texture features, then employed decision trees to classify six types of acne lesions, achieving 85.5% accuracy. Alamdari et al. (86) implemented a mobile application that segments lesions via k-means and classifies them with fuzzy logic and SVMs, reporting 100% accuracy in acne detection and up to 80% in scar classification. Kittigul and Uyyanonvara (87) extracted speeded-up robust features (SURF) descriptors along with hue mean, RGB (Red, Green, Blue) standard deviations, and circularity, and used a k-nearest neighbors (k-NN) classifier to achieve 73% sensitivity, 84% precision, and 68% overall accuracy. Al-Tawalbeh et al. (88) built a three-class skin-lesion classifier (benign, melanoma, seborrheic keratosis) using 71 color and texture features across multiple color spaces and Gabor filters; a second-order polynomial SVM yielded 95.8% overall accuracy and 99.7% precision for seborrheic keratosis on non-segmented images. Finally, Maroni et al. (89) combined Haar-cascade body-part detection with random-forest skin segmentation (using multimodal features), CIELab heat-mapping, adaptive thresholding, and Laplacian-of-Gaussian blob detection to count acne lesions and monitor severity under real-world conditions.

As shown in Table 5, traditional machine learning approaches can be broadly classified into unsupervised and supervised methods. Unsupervised techniques (e.g., saliency-based clustering and fuzzy C-means) require no labeled data and offer automated segmentation and measurement, but they can be sensitive to parameter choices and imaging variation. In contrast, supervised methods (e.g., LBP + SVM, SURF + k-NN, Gabor + multiclass SVM) learn from annotated examples to achieve higher classification accuracy, though their performance depends heavily on the quality and diversity of the training dataset.

**Table 5:** Traditional machine learning methods for scar recognition and diagnosis.

**References**	**Method description**	**Data**	**Metrics**	**Supervised?**
Ma et al. (79)	Saliency detection using Gaussian pyramid and clustering	–	–	Unsupervised
Liu et al. (83)	LBP & wavelet features, SVM classification	Microscopy images	–	Supervised
Jiang et al. (82)	Smartphone-based 3D modeling and measurements	33 keloids, 28 patients	ICC > 0.95	Unsupervised
Heflin et al. (84)	Scar/tattoo detection in uncontrolled images	Forensic scenarios	–	Supervised
Chantharaphaichi et al. (81)	Rule-based acne detection	Acne lesion images	High sensitivity; prone to false positives	Unsupervised
Khan et al. (80)	Fuzzy C-means clustering	50 acne images	Accuracy: 92.63%	Unsupervised
Abas et al. (85)	Entropy-based ROI, decision tree classification	Acne images	Accuracy: 85.5%	Supervised
Alamdari et al. (86)	K-means segmentation + fuzzy/SVM	Acne/scar images	Acne: 100%, Scar: 80%	Supervised
Kittigul and Uyyanonvara (87)	SURF feature extraction, k-NN classification	Acne images	Sens: 73%, Prec: 84%, Acc: 68%	Supervised
Al-Tawalbeh et al. (88)	71 color/texture features, polynomial SVM	Skin lesion images	Acc: 95.8%; Prec: 99.7% (SK)	Supervised
Maroni et al. (89)	Haar-cascade + RF skin segmentation + blob detection	Smartphone acne images	Lesion counting and severity tracking	Supervised

### 6.5 Unsupervised CNN-based methods

To date, there have been no purely unsupervised CNN architectures applied to scar recognition or diagnosis in the literature covered; all deep-learning approaches rely on annotated data and end-to-end supervised training to learn feature representations or perform segmentation. However, recent work has explored deep reinforcement learning (DRL) as a form of weakly supervised segmentation that does not require pixel-wise annotation during inference. Usmani et al. (90) cast lesion delineation as a Markov decision process and train an agent via deep deterministic policy gradient (DDPG) to “draw” segmentation masks in a continuous action space, using only global reward signals derived from expert-provided ground-truth masks. Their method achieved accuracy of 96.33% on naevus, 95.39% on melanoma, and 94.27% on seborrheic keratosis in the ISIC 2017 dataset, and comparable performance on HAM10000 and PH2 (96.3%, 95.4%, and 94.3%, respectively). Although this approach still depends on ground-truth masks to compute rewards during training, it eliminates the need for dense, step-by-step pixel annotations and thus represents a promising direction toward unsupervised—or more accurately, weakly supervised—deep segmentation methods for skin lesions.

### 6.6 Supervised CNN-based methods

**Figure 9:**
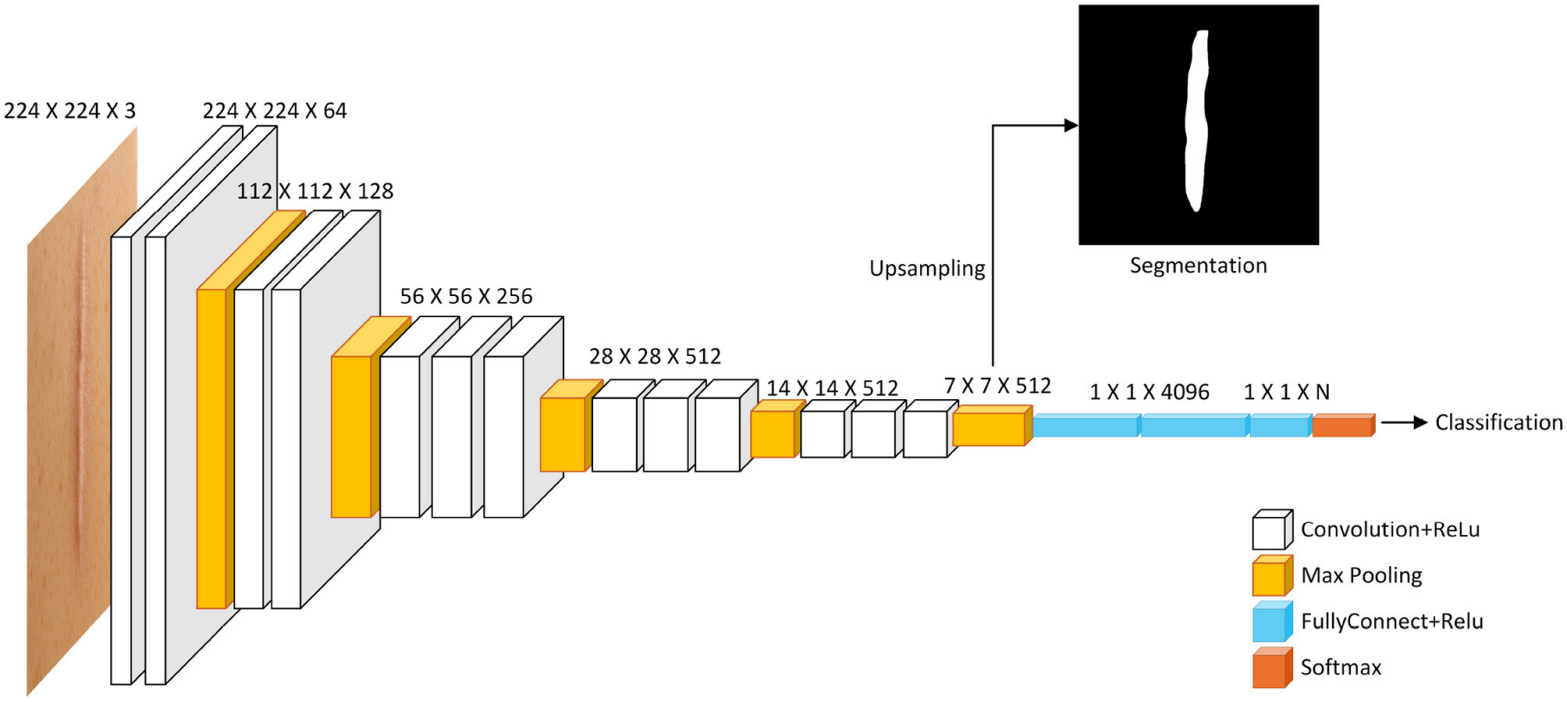
Diagram illustrating a deep learning model structure for image processing. It shows layers including Convolution and ReLU, Max Pooling, Fully Connected with ReLU, and Softmax. The input is a 224 x 224 x 3 image. Outputs include a segmentation image through upsampling and a classification result. Layer dimensions decrease from left to right, with specified sizes at each stage. A multi-task CNN architecture based on VGG. The shared convolutional backbone feeds into two branches: one for image classification via fully connected layers, and another for semantic segmentation. The segmentation path requires upsampling (e.g., transposed convolution) to restore spatial resolution.

Convolutional neural networks (CNNs) have rapidly become the state of the art in scar recognition and diagnosis by learning hierarchical feature representations directly from data. Table 6 summarizes key CNN architectures, datasets, and performance metrics reported in the literature, while Figure 9 illustrates a prototypical multi-task VGG-based network that combines classification and segmentation branches. In the following section, we examine these supervised CNN approaches by discussing their design innovations, clinical datasets, and quantitative outcomes, and then outline the challenges they face in generalization, interpretability, and computational demands.

**Table 6:** CNN-based methods for scar recognition and diagnosis.

**References**	**Method description**	**Data**	**Metrics**
Usmani et al. (90)	Deep RL-based lesion segmentation via DDPG	ISIC 2017, HAM10000, PH2	Acc: 96.3% (nevus), 95.4% (melanoma), 94.3% (seborrheic keratosis)
Pham et al. (91)	Modified VGG-16 CNN	Histology images	Accuracy: >97%
Pham et al. (92)	Universal CNN model	General histology	High robustness
Maknuna et al. (93)	Mask R-CNN, K-means	Scar images	Effective collagen characterization
Chu et al. (94)	ResNet-50 CNN, MCPL	7,524 images, 3,565 patients	AUROC: 0.915
Kim et al. (78)	CNN-based severity prediction	1,283 patients	Comparable accuracy to dermatologists
Junayed et al. (95)	ScarNet (19-layer CNN)	Acne scar images	Optimized accuracy, efficient
Ito et al. (96)	CNN-based scar classification	Clinical images	Accuracy: 77% (Doctors: 68.7%)
Singh and Saxena (97)	CNN for collagen structure	Treatment efficacy images	Efficacy evaluation
Privalov et al. (98)	Mask R-CNN segmentation	Wound photographs	Effective segmentation
Rajesh et al. (99)	Custom CNN for vitiligo/scars	3135 images	Accuracy: 93.89%, Precision: 96.5%, AUC: 0.95
Abdolahnejad et al. (100)	EfficientNet B7, segmentation, K-means	6,550 images, longitudinal	Accuracy: 98%, ± 2 mm error
Aguilar et al. (101)	CNN acne scar risk assessment	437 images, 404 patients	Accuracy: 93.15%, AUC: 0.931

**Figure 10:**
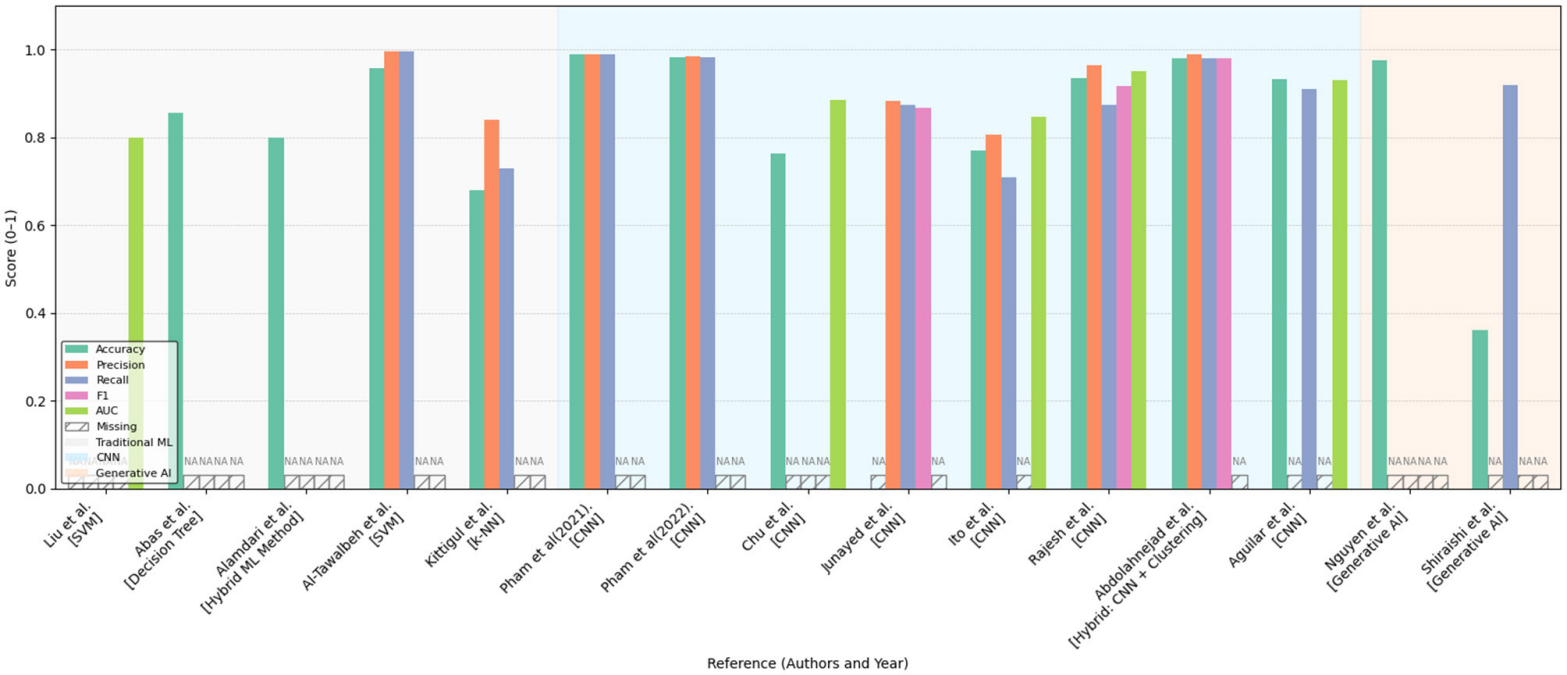
Bar chart comparing various models and methods in terms of accuracy, precision, recall, and F1 score. Different colors represent different metrics. Some bars have data labeled as “NA,” indicating missing information for certain methods. Categories include traditional ML, hybrid ML, GNN, and generative AI. Performance comparison of various machine learning and deep learning approaches across multiple evaluation metrics (Accuracy, Precision, Recall, F1-score, and AUC) as reported in existing literature. The methods are grouped by model type: traditional machine learning (gray-shaded background), convolutional neural networks (light blue background), and generative AI models (light orange background). Missing values are indicated as “NA.” The figure highlights the relative strengths and limitations of each approach within a normalized score range (0–1), providing a comprehensive overview of their effectiveness in the reviewed studies.

**Figure 11:**
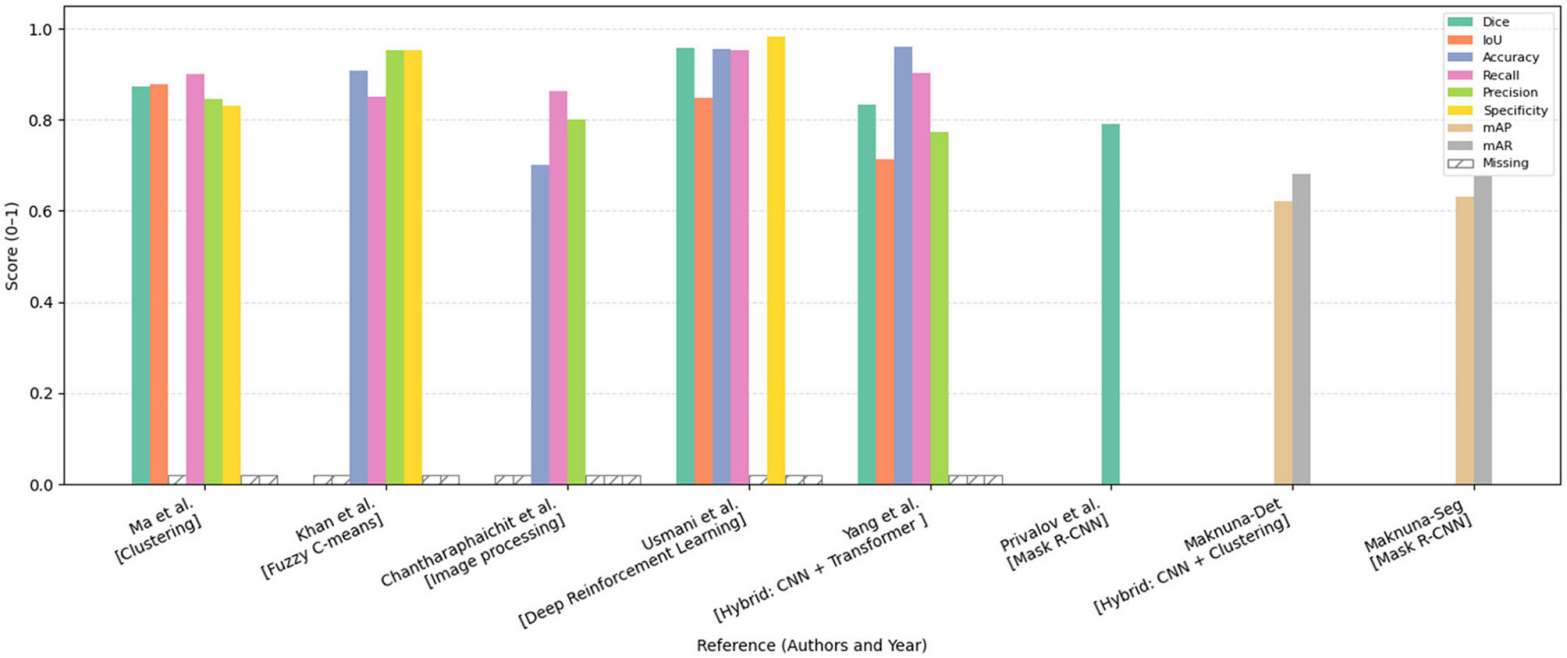
Bar chart comparing the performance of various methods, including clustering, fuzzy C-means, image processing, deep reinforcement learning, CNN, and Mask R-CNN. The chart displays scores across metrics Dice, IoU, accuracy, recall, precision, specificity, mAP, and mAR. Each category has colored bars representing these metrics, with most scores ranging from 0.8 to 1.0. The missing data is marked with a hatch pattern. Comparative performance analysis of segmentation and detection methods reported in this chapter, evaluated across various metrics including Dice coefficient, Intersection over Union (IoU), Accuracy, Recall, Precision, Specificity, mean Average Precision (mAP), and mean Average Recall (mAR). The models are categorized by methodological approach. Missing metric values are denoted as “Missing.” All scores are normalized within the range [0, 1], facilitating a standardized comparison of model effectiveness across diverse approaches.

Figures 10, 11 summarize the performance metrics of the methods discussed in this chapter, corresponding to classification and segmentation tasks, respectively. These visual comparisons aim to provide readers with a convenient overview of how different approaches perform under commonly used evaluation criteria. The figures include only studies that report standard quantitative metrics; methods employing less conventional evaluations (e.g., mask dimension differences in segmentation) are not represented. Similarly, studies focusing on tasks such as scar characterization or analysis are excluded, as these often lack universally adopted quantitative benchmarks.

Due to differences in datasets, sample sizes, and experimental protocols, the reported metrics should be interpreted with caution. Readers are advised to consult the preceding tables and method descriptions for contextual understanding. For studies evaluated on multiple datasets, the plotted results reflect weighted averages based on dataset size to ensure consistent comparison.

In the following, we review these supervised CNN approaches—highlighting their design innovations, clinical datasets, and quantitative outcomes—as well as the challenges they face in generalization, interpretability, and resource demands.

Pham et al. (91) developed a deep learning-based method using a modified VGG-16 CNN to classify and quantify collagen fiber organization in burn-induced scar tissue from Masson's Trichrome (MT)-stained histology images. The model achieves over 97% classification accuracy and effectively extracts collagen density and directional variance, revealing significant structural differences between scar and normal tissue. While demonstrating robustness across multi-scale images, limitations include sensitivity to tissue heterogeneity and restriction to MT staining. Afterwards, they further proposed a universal CNN model that does not rely on specific histological staining processes to classify and characterize collagen fiber structures in burn-induced scar tissue (92). Maknuna et al. (93) employed machine learning techniques for the automated structural analysis and quantitative feature description of scar tissue. Using Mask R-CNN and K-means algorithms, the study effectively predicted and characterized scar images, such as collagen density and directional variation. Chu et al. (94) proposes a deep learning-based approach for the classification of post-thyroidectomy scar subtypes using a ResNet-50 CNN and a novel multiple clinical photography learning (MCPL) method. A dataset of 7,524 clinical photographs from 3565 patients was used to train and validate the model. The MCPL method, which leverages multiple images of the same scar per patient, improved model robustness and classification accuracy compared to a baseline model, achieving an AUC of up to 0.915 for hypertrophic scars. Kim et al. (78) developed an AI model to predict the severity of post-surgical scars. Using data from 1,283 patients (1,043 in the main dataset and 240 in the external dataset), the model demonstrated comparable accuracy to that of 16 dermatologists. Junayed et al. (95) developed a deep CNN model named ScarNet for the automatic classification of acne scars. ScarNet employs a 19-layer deep learning architecture, with optimizations made to the activation functions, optimization algorithms, loss functions, kernel sizes, and batch sizes to improve classification performance while reducing computational costs. Ito et al. (96) developed a computer vision algorithm based on automated machine learning for diagnosing four types of scars: immature scars, mature scars, hypertrophic scars, and keloids. Compared to doctors' diagnoses, the algorithm achieved an average accuracy of 77%, while doctors' average accuracy was 68.7%. Singh and Saxena (97) developed an image processing algorithm using CNN to evaluate treatment efficacy by analyzing collagen fiber structures in scar images. Privalov et al. (98) validated an automated wound segmentation and measurement method based on Mask R-CNN for processing wound photographs. Rajesh et al. (99) proposes a deep learning-based approach for classifying vitiligo and scar images using a customized CNN with six convolutional layers and three fully connected layers. A dataset of 3,135 images was used, augmented to improve generalization. The model achieved a training accuracy of 93.89%, precision of 96.50%, and an AUC score of 0.95, outperforming existing architectures such as ResNet-50, InceptionV3, and VGG-16. Abdolahnejad et al. (100) introduces a machine learning pipeline for the automated assessment and longitudinal tracking of keloid scars, integrating EfficientNet B7-based CNN for classification, segmentation techniques for lesion boundary detection, and K-Means clustering for colorimetric analysis. The model was trained on a dataset of 6,550 images, achieving a classification accuracy of 98%, with segmentation refined using fiducial markers and contour-based detection. The pipeline was validated through 5–6 months of follow-up imaging, effectively capturing changes in keloid size and pigmentation with a measurement error margin of ± 2 mm. Despite its high accuracy, the method demonstrated limitations in detecting early-stage keloids and challenges in segmenting lesions on darker skin tones due to reduced contrast. Aguilar et al. (101) explores the feasibility of using CNN for automated acne scar risk assessment. A dataset of 437 clinical images from 404 acne patients was annotated by dermatologists and categorized using the four-item Acne-Scar Risk Assessment Tool (4-ASRAT) into low-, moderate-, and high-risk groups. A custom CNN model was trained for both binary (risk/no risk) and three-class classification, achieving 93.15% accuracy and an AUC of 0.931 for the binary classification task. However, performance on the three-class classification was poor (68.26% accuracy) due to the lack of clear separation between mild and severe scarring categories.

### 6.7 Key limitations and failure modes of CNN-based diagnostic models

Convolutional neural networks (CNNs) have emerged as the cornerstone of numerous state-of-the-art diagnostic systems, offering remarkable performance improvements over traditional machine learning and rule-based methods. Their ability to automatically learn hierarchical representations from raw medical images has led to substantial gains in tasks such as disease classification, lesion detection, and image segmentation. Particularly in domains like radiology, dermatology, and ophthalmology, CNN-based models have approached or even exceeded expert-level diagnostic accuracy in controlled settings. Furthermore, CNNs are highly adaptable to diverse imaging modalities (e.g., CT, MRI, histopathology), and benefit from transfer learning, making them broadly applicable across medical subfields.

However, despite these strengths, CNN-based diagnostic models are not without significant limitations. Their practical deployment in clinical environments is hindered by a series of non-trivial challenges, which compromise model robustness, reliability, and trustworthiness. Below, we summarize key failure modes and systemic limitations of CNNs in biomedical applications.

#### 6.7.1 Overfitting and limited generalization in small clinical datasets

**Figure 12:**
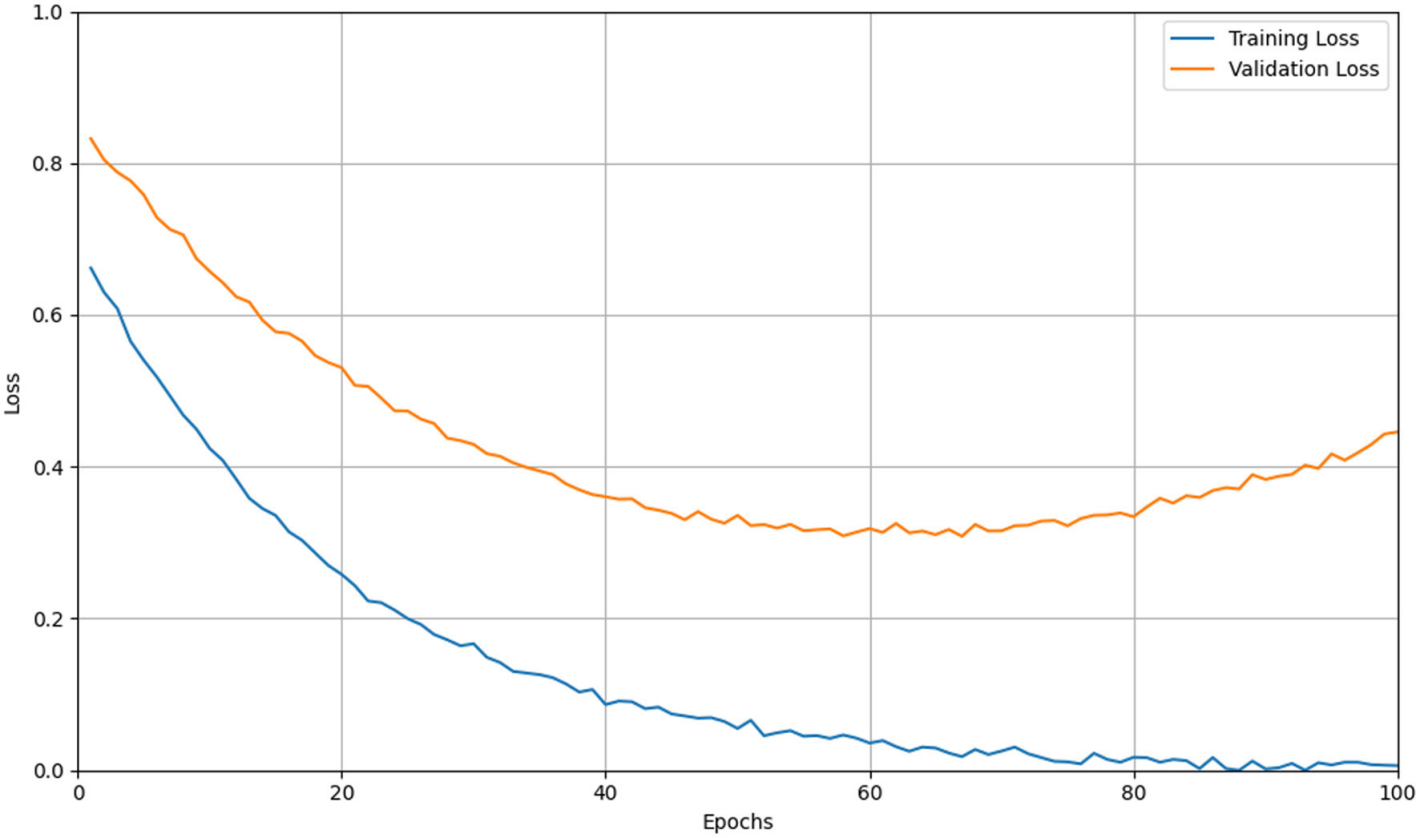
Line graph showing training and validation loss over 100 epochs. Training loss (blue) decreases steadily, while validation loss (orange) decreases initially but rises after 50 epochs, indicating potential overfitting. Simulation of overfitting in CNN training on limited data. The yellow curve traces the training loss steadily decreasing, while the orange curve shows the validation loss initially falling but then rising after mid-training. The divergence between these curves indicates the model's tendency to memorize training-specific noise and lose generalization capability as epochs progress.

While CNNs excel at learning from large-scale annotated corpora, clinical datasets are often limited in size and suffer from class imbalance, institutional bias, and acquisition variability. This mismatch between model capacity and data availability can lead to overfitting, where CNNs memorize dataset-specific artifacts rather than learning disease-relevant features, As shown in Figure 12. Despite the architectural regularization imposed by convolutional layers and weight sharing, CNNs still require extensive regularization strategies—such as data augmentation, dropout, weight decay, transfer learning, and early stopping—to mitigate this issue and improve generalization to unseen data (102, 103).

#### 6.7.2 Vulnerability to adversarial perturbations and distributional shifts

CNN-based diagnostic systems are highly susceptible to adversarial attacks—minute, often imperceptible modifications to input images that can drastically alter model predictions. Finlayson et al. (104) demonstrated that adversarial examples could significantly impair CNN performance across multiple medical domains under both white-box and black-box threat models. Moreover, CNNs often fail to maintain accuracy when exposed to distributional shifts, such as changes in imaging protocols, hardware, or patient demographics. Recent studies have emphasized the need for robust training paradigms, including adversarial training, domain adaptation, and input validation, to ensure model reliability under real-world deployment scenarios (105, 106).

#### 6.7.3 Sensitivity to batch size and optimization-induced generalization gaps

**Figure 13:**
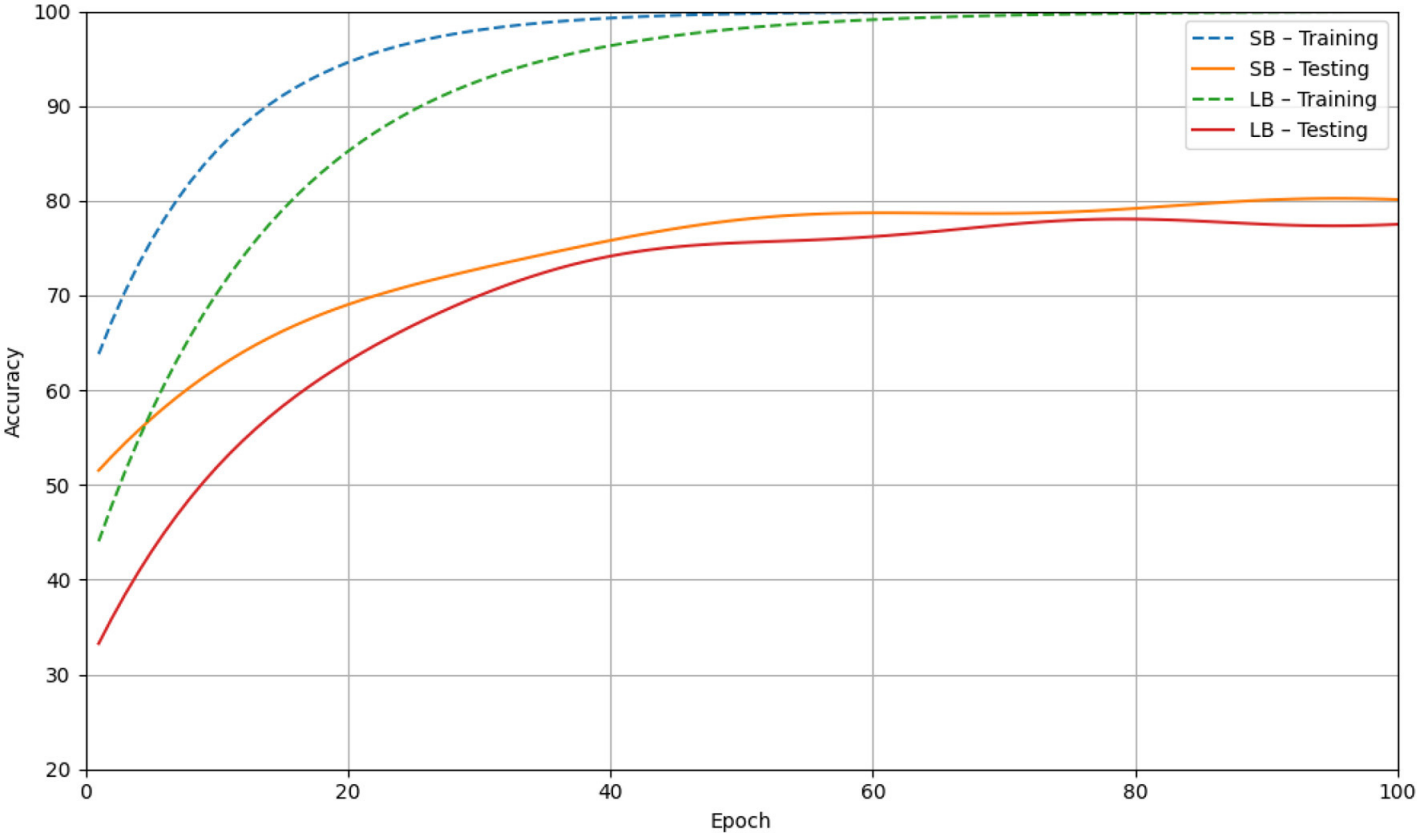
Line graph depicting accuracy over epochs for training and testing sets. SB-Training in dashed blue and SB-Testing in solid orange both increase steeply, reaching over 95 and 75 respectively. LB-Training in dashed green and LB-Testing in solid red increase more gradually, reaching about 90 and 70 respectively. Simulation of batch-size sensitivity in CNN training on CIFAR-10. Dashed lines represent training accuracy for small-batch (SB, blue) and large-batch (LB, red) regimes, while solid lines show the corresponding test accuracy. The chart illustrates that larger batch sizes yield reduced gradient noise, converge to sharper minima, and exhibit a substantially wider generalization gap compared to smaller batches.

Optimization dynamics in CNN training are significantly influenced by batch size. Keskar et al. (107) showed that large-batch (LB) training (e.g., >1,000 samples) tends to converge to sharp local minima in the loss landscape, which are associated with poor generalization performance. In contrast, small-batch (SB) training (e.g., 32–128 samples) introduces stochasticity that encourages convergence to flatter minima, yielding more robust models, as shown in Figure 13. Masters and Luschi (108) further demonstrated that extremely small batches (as few as 2–32 samples) often achieve the best generalization even on large datasets like ImageNet and CIFAR-10/100. This highlights the importance of tuning batch size as a hyperparameter and considering its interaction with learning rate schedules in biomedical applications.

#### 6.7.4 Lack of interpretability and opaqueness of decision processes

The black-box nature of CNNs raises major concerns in high-stakes domains such as medicine. While techniques like Grad-CAM and other saliency-based methods attempt to visualize decision rationales, they often produce inconsistent or misleading explanations—e.g., focusing on irrelevant regions, highlighting only dominant lesions in multi-lesion cases, or failing basic sanity checks like weight randomization and reproducibility tests (109, 110). This lack of reliable interpretability undermines clinical trust, impairs model debugging, and complicates regulatory approval, emphasizing the need for more principled and faithful explanation methods.

#### 6.7.5 Summary and future directions

In summary, while CNN-based models have revolutionized image-based diagnosis through automated feature learning and superior classification performance, their real-world clinical utility remains constrained by several critical limitations. These include susceptibility to overfitting on small and heterogeneous clinical datasets, vulnerability to adversarial perturbations and domain shifts, sensitivity to training dynamics such as batch size, and persistent issues surrounding interpretability and transparency. Such failure modes not only challenge the robustness of CNNs but also raise ethical and regulatory concerns, particularly in safety-critical applications.

Addressing these challenges calls for a multifaceted research agenda. On the data side, collaborative efforts to curate large-scale, diverse, and well-annotated medical image repositories—ideally spanning multiple institutions and patient demographics—are essential to improve generalization and fairness. In terms of algorithmic development, future work should prioritize robust optimization strategies that are resilient to data shifts and adversarial noise, such as distributionally robust learning, self-supervised pretraining, and uncertainty-aware inference. Furthermore, integrating domain knowledge (e.g., anatomical priors or clinical guidelines) into model design may offer inductive biases that enhance generalizability and interpretability.

Finally, the development of inherently interpretable CNN architectures and rigorous *post hoc* explanation tools remains a pressing need. These efforts should be coupled with standardized benchmarks and clinical evaluation protocols to quantify explanation reliability and diagnostic value. Bridging the gap between algorithmic performance and clinical trustworthiness will be vital to transition CNN-based diagnostic systems from promising prototypes to dependable tools in routine medical practice.

### 6.8 Other advanced methods

#### 6.8.1 Methods combining 3D reconstruction and deep learning

integrating 3D reconstruction with deep learning techniques allows for comprehensive analysis and quantification of scars in three-dimensional space, particularly beneficial for precise measurements and assessments on complex skin surfaces. The combination of 3D modeling with deep neural networks opens new avenues for highly accurate, non-contact scar evaluation. Below we discuss recent developments in this promising research direction, and relevant references are also listed in Table 7.

**Table 7:** Advanced methods combining 3D reconstruction, deep learning, and foundation models for scar diagnosis.

**References**	**Method description**	**Dataset/scale**	**Performance metrics**
Wang et al. (111)	SHG imaging, GAN, CNN regression	SHG imaging data	Scar collagen texture quantification
Zhou et al. (112)	3D reconstruction, CNN segmentation	Smartphone forensic images	Measurement error: 3.69%
Zhou et al. (113)	3D reconstruction, CNN segmentation with augmentation	Multi-view scar images	Improved segmentation accuracy
Yan et al. (64)	Multimodal foundation model (PanDerm) pretrained via self-supervised learning	Over 2 million dermatological images	Superior accuracy over clinicians; significant data efficiency
Nguyen et al. (120)	ChatGPT-based image analysis model predicting scar outcomes	40 plastic surgery patients	Binary classification accuracy: 97.5%; width prediction *R*^2^ = 0.956; height prediction *R*^2^ = 0.857
Shiraishi et al. (121)	ChatGPT-based models differentiating keloids from hypertrophic scars	30 clinical scar images	GPT-4 accuracy: 36.0%; Specificity (keloid: 0.60, hypertrophic scar: 0.72)
Yang et al. (122)	Swin Transformer and CNN dual encoder + MFFM and multi-pooling channel-spatial attention mechanism	265 clinical scar images	Acc: 96.01%; Prec: 77.43%; Dice: 83.21%

Wang et al. (111) proposed a novel method combining second harmonic generation (SHG) imaging technology and deep learning algorithms. By integrating SHG imaging with GAN and utilizing Tamura texture features, they constructed a regression model to quantitatively analyze collagen textures in human scar tissue and predict scar development. Zhou et al. (112) proposes a deep learning-based method for the automatic measurement of linear scar lengths, particularly for forensic applications. By integrating multiview stereo 3D reconstruction and CNN for image segmentation, the method allows non-contact, automated, and high-accuracy scar measurement using images taken from a smartphone. The model achieved an average measurement error of 3.69%, demonstrating strong agreement with manual measurements. Compared to traditional manual and 2D imaging methods, this approach reduces subjectivity and improves accuracy, especially for scars on curved surfaces. However, limitations include time-consuming 3D reconstruction and reliance on training data quality. Future research should optimize computational efficiency, improve segmentation models, and explore broader clinical applications. Zhou et al. (113) proposed an advanced two-stage deep learning framework for scar segmentation in multi-view images. The first stage includes a novel data augmentation method based on 3D reconstruction and view interpolation to enhance the model's generalization ability.

Methods combining 3D reconstruction with deep learning show substantial promise in enhancing measurement precision and automated analysis of scars, particularly in forensic and clinical contexts requiring high accuracy. Despite their benefits, the significant computational cost, complexity of data acquisition, and reliance on high-quality training data are notable limitations requiring further exploration.

#### 6.8.2 Computational footprint of 3D-reconstruction-driven scar assessment

Although 3D reconstruction noticeably improves geometric fidelity, its clinical roll-out hinges on practical runtime and hardware demands. Table 8 summarizes the details of computational resource requirements for these research works. Wang et al. (111) needed roughly six minutes per case on unspecified hardware, with ScarGAN accounting for most of the 360 s pipeline latency. Zhou et al. (112) executed on a consumer-grade RTX 2060 (8 GB) workstation; even after aggressive image down-sampling, structure-from-motion took a mean ± Standard Deviation (SD) of 111.8 ± 19.9 s, while subsequent measurement added 28.1 ± 8.4 s. To cope with heavier multi-view co-segmentation, Zhou et al. (113) upgraded to an RTX 3090 (24 GB); their MVCSNet contains 31.0 M parameters and incurs 218.7 GFLOPs per forward pass—no end-to-end runtime was disclosed, but the authors note that GPU memory constrained batch-size to 1.

**Table 8:** Reported computational profile of 3-D reconstruction methods for scar analysis.

**Study**	**Hardware (GPU/CPU)**	**Time per sample (s)**	**Params (M)**	**FLOPs (G)**
Wang et al. (111)	–	360	–	–
Zhou et al. (112)	RTX 2060 8 GB + i7-9700	111.8 (recon) + 28.1 (measure)	–	–
Zhou et al. (113)	RTX 3090 24 GB	–	31.0	218.7

Collectively, these data indicate that current 3-D Reconstruction methods still require **30s–6min per patient and 8–24 GB of GPU memory**–tolerable for retrospective analysis yet insufficient for real-time clinical use. Future work should (i) publish full training and inference profiles, (ii) apply pruning, quantisation, and mixed-precision to cut memory below 4 GB, and (iii) replace global multi-View stereo (MVS) with lightweight depth-fusion schemes to bring per-case runtime under 10 s.

#### 6.8.3 Large-scale foundation models in scar diagnosis

**Figure 14:**
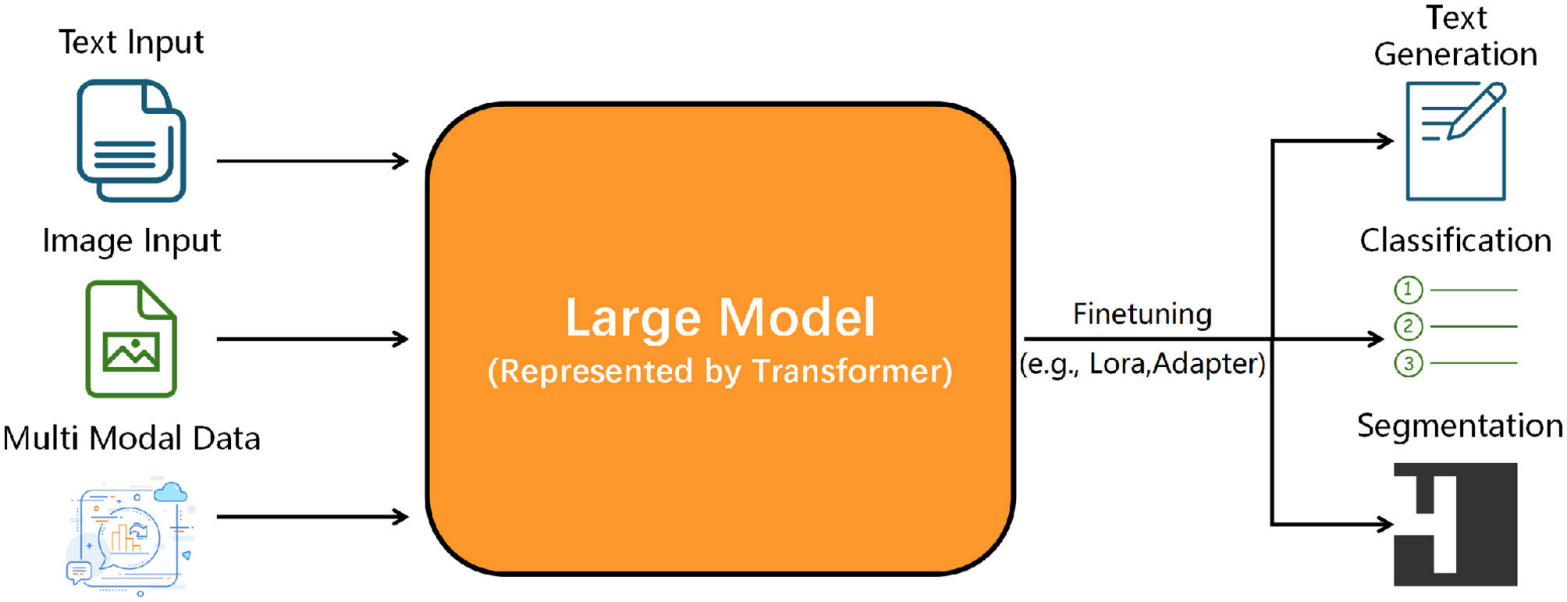
Diagram of a large model represented by a transformer. Inputs include text, image, and multimodal data, with outputs for text generation, classification, and segmentation. Finetuning options like LoRa and Adapter are shown. A generic architecture of large-scale foundation models. The model accepts various types of input (e.g., text, image, or multimodal data), processes them through a unified backbone—typically based on Transformer architecture—and supports a range of downstream tasks such as text generation, classification, and segmentation. Lightweight finetuning methods (e.g., LoRA, adapters) can be employed to adapt the model to specific applications.

Recent advancements in foundation models have demonstrated considerable potential in dermatological diagnostics, including scar recognition and evaluation. A prominent work by Yan et al. (64), published in *Nature Medicine*, introduced PanDerm, a multimodal vision foundation model pretrained via self-supervised learning on over two million dermatological images collected from 11 clinical institutions, encompassing clinical photography, dermoscopic images, total-body photography, and dermatopathological slides. A general architecture of such large-scale foundation models is illustrated in Figure 14. PanDerm achieved state-of-the-art performance across diverse clinical tasks, notably demonstrating superior data efficiency by surpassing existing methods even when utilizing only 10% of labeled data. Clinical validation confirmed PanDerm's substantial clinical value, notably outperforming clinicians by 10.2% in early-stage melanoma detection and improving diagnostic accuracy across 128 skin conditions by 16.5% among non-specialists. This seminal work highlights the transformative potential of multimodal foundation models in comprehensive dermatological assessments, providing a critical reference point for future intelligent scar diagnostics.

PanDerm has demonstrated broad applicability across dermatological tasks through pretraining on over two million multimodal skin disease images. However, fine-tuning for scar evaluation requires addressing the unique morphological, chromatic, and vascular features of scar tissue. First, it is essential to curate a high-quality dataset that includes dermoscopy, optical coherence tomography, and ultrasound images with precise annotations of scar width, height, vascularity, and pigmentation. In the fine-tuning phase, parameter-efficient methods such as layer-wise differentiation of learning rates (freezing early convolutional and Transformer layers while fully training later layers) and Low-Rank Adaptation (LoRA) can significantly reduce trainable parameters without sacrificing performance (114, 115). Moreover, integrating multi-task objectives for scar segmentation, classification, and regression within a shared backbone exploits cross-task synergies, as shown by self-training frameworks leveraging confident pseudo-labels for segmentation (116). To mitigate domain shift between PanDerm's broad pretraining domain and the scar-specific target domain, adversarial domain adaptation techniques such as Domain-Adversarial Neural Networks with gradient reversal layers can promote extraction of scar-invariant features and improve generalization across clinical centers (117, 118). When annotation resources are limited, pseudo-label self-training can expand the training corpus by using confident predictions on unlabeled images. Finally, applying self-supervised pretraining strategies such as masked image modeling or contrastive multimodal learning on scar-centric datasets can further regularize the model, reduce overfitting, and pave the way for federated and few-shot scar assessment systems that support privacy-preserving deployment in diverse clinical settings (119).

Other explorations have investigated generative AI frameworks in scar prognosis and classification. Nguyen et al. (120) assessed the feasibility of a ChatGPT-integrated image analysis model in predicting long-term scar characteristics. Evaluating standardized images from 40 plastic surgery patients, the ChatGPT-based approach achieved remarkable accuracy (97.5%) for binary scar classification. The model performed exceptionally well in predicting static scar attributes such as width (*R*^2^ = 0.956) and height (*R*^2^ = 0.857), although dynamic features, such as vascularity (*R*^2^ = 0.234) and pigmentation (*R*^2^ = 0.676), remain challenging. These findings highlight the promising yet currently limited capability of generative AI for objective, long-term scar prediction, especially concerning dynamic scar properties.

Additionally, Shiraishi et al. (121) explored the potential of ChatGPT-based models in distinguishing between keloids and hypertrophic scars through standardized clinical image prompts. Comparing multiple AI chatbots, GPT-4 significantly outperformed others, achieving a higher diagnostic accuracy (36.0% vs. 22.0%) and notably better specificity. Nevertheless, current generative AI models still fall short of clinical standards, underscoring the need for further refinements in accuracy and robustness. This preliminary work provides valuable insights into the potential and limitations of applying large language models for scar diagnosis.

While the above research works exemplifies the potential of large-scale, general-purpose foundation models, task-specific architectures leveraging key components of such models-particularly Transformers-have also shown notable promise. For instance, Yang et al. (122) proposed MFMA-Net, a dual-encoder segmentation network that integrates a CNN and a Swin Transformer to capture both local textures and global context. Through a multi-scale feature fusion module and a multi-pooling channel-spatial attention mechanism, the model achieved state-of-the-art performance on clinical scar segmentation tasks (e.g., 96.01% accuracy, 83.21% Dice coefficient), outperforming classical and Transformer-based baselines alike. Though MFMA-Net is not a foundation model *per se*, it demonstrates how Transformer-based designs can be effectively adapted for high-precision, task-specific applications in scar assessment.

It is worth noting that, to date, only the aforementioned study has specifically applied Transformer-based or foundation model-inspired architectures to scar diagnosis. However, similar advanced artificial intelligence techniques have been extensively explored in related domains, including the detection, segmentation, and classification of skin lesions and skin cancer (123–149). Beyond these dermatological applications, other biomedical-image analysis tasks have also benefited from novel Transformer architectures. Xiang et al. (150) propose a two-stage Multimodal Masked Autoencoder (Multi-MAE) for vitiligo stage classification. The approach integrates an adaptive masking module that leverages self-attention to dynamically mask and discard non-salient patches, a unified Vision Transformer encoder shared by clinical and Wood's lamp images, and a cross-attention fusion decoder for multimodal reconstruction pre-training. On a modest multimodal dataset, Multi-MAE achieved 95.48% accuracy, outperforming MobileNet, DenseNet, VGG, ResNet-50, BEIT, MaskFeat, SimMIM, and standard MAE by 2.58%–5.16%. Similarly, Song et al. (151) introduce CenterFormer, an end-to-end transformer-based framework for unconstrained dental plaque segmentation. It features a Cluster Center Encoder (CCE) that applies K-means clustering on multi-level feature maps to produce coarse region representations, a pyramid-style Multiple Granularity Perceptions module to fuse local and global contexts, and an MLP decoder for final mask prediction. Evaluated on nearly 3,000 intraoral images, CenterFormer attained an IoU of 60.91% and a pixel accuracy of 76.81%, surpassing SegFormer and other state-of-the-art models by 2.34%–6.08%. Although tailored to vitiligo staging and dental imagery, respectively, both methods employ adaptive, self-supervised pre-training, multimodal fusion, clustering-guided attention, and multi-scale feature integration strategies that are methodologically relevant and technically transferable to scar-related tasks. We expect that such techniques will play an increasingly important role in future developments of intelligent scar analysis.

### 6.9 Clinical integration

Technical accuracy alone is insufficient to deliver patient-centered value in scar care. Given the documented inter-rater variability of visual/tactile scales, AI systems should be embedded across the care pathway with explicitly defined links to psychosocial wellbeing and satisfaction. Below we outline practical integration points, implementation guardrails, and evaluation strategies, drawing on evidence from dermatology and other clinical domains.

#### 6.9.1 Pre-visit intake and education

Smartphone-guided image capture with automated quality control can reduce uncertainty before clinic visits, while brief electronic patient-reported outcomes (ePROs)-for example, POSAS-patient items, itch/pain numerical rating scales, and short dermatology quality-of-life screens-establish a baseline and flag high-risk psychosocial profiles for clinician review. Educational feedback (expectation setting; capture tips for darker skin tones and various illumination conditions) may lower anxiety and improve perceived preparedness. Teledermatology workflows that combine images with structured questionnaires have shown high patient satisfaction and shorter time-to-advice in multiple evaluations (152, 153).

#### 6.9.2 In-clinic decision support and shared decision-making

AI-aided segmentation, severity scoring, and progression/recurrence risk prediction can be surfaced within the electronic record to support share decision-making. Calibrated risk displays, standardized visual aids (e.g., 3D surface metrics), and curated exemplar galleries help align expectations, reduce decisional conflict, and personalize plans (e.g., prophylaxis for keloid-prone patients). Experience from other specialties (e.g., autonomous diabetic retinopathy screening deployed in primary care) illustrates how validated AI can be safely integrated into routine pathways with defined scopes of use, audit trails, and referral rules (154, 155).

#### 6.9.3 Longitudinal follow-up and remote monitoring

Between visits, remote monitoring can pair periodic photos with short ePROs to track trajectory and symptoms (itch, pain, appearance concerns, sleep impact). Drift-aware alerts notify teams of worsening objective metrics or deteriorating ePROs and can trigger timely intervention or referral to psychological support. Evidence from ePRO programs in other fields shows that structured symptom monitoring can improve quality of life and care experience, and in some settings clinical outcomes (156).

#### 6.9.4 Implementation guardrails and equity

Deployment should adopt human-in-the-loop oversight, clear scope-of-use statements, fail-safes for uncertainty, and governance for updates. Equity safeguards include validated performance across skin tones, scar architectures, and acquisition settings, plus multilingual, accessible capture instructions. Integration with EHRs (e.g., via HL7 FHIR) should store AI outputs, timestamps, and ePROs with audit trails. Privacy and consent must explicitly cover image content and per-image metadata, consistent with international guidance on trustworthy AI in health (157, 158).

In sum, integrating AI into routine scar care can translate technical accuracy into patient-centered value. Embedding decision support at pre-visit, in-clinic, and follow-up stages reduces uncertainty, aligns expectations, and enables timely escalation, while remote monitoring pairs objective image metrics with brief ePROs to track psychosocial needs. Human-in-the-loop oversight, clear scopes of use, and privacy-by-design safeguard safety and trust. Ultimately, success should be judged not only by diagnostic performance but also by improvements in patient-reported outcomes, adherence, and satisfaction, demonstrating tangible clinical benefit.

## 7 Conclusion

This review systematically summarized and analyzed recent advancements in intelligent recognition and diagnosis methods for skin scars, encompassing traditional machine learning methods, convolutional neural network (CNN)-based approaches, and hybrid methods integrating 3D reconstruction and deep learning. Traditional clinical scar assessment methods, although valuable in clinical practice, inherently suffer from subjectivity and inconsistencies arising from varied expertise levels among clinicians (159). In contrast, intelligent diagnostic methods leveraging artificial intelligence (AI) provide objective, reproducible, and efficient tools that can significantly enhance clinical assessments and patient care.

Traditional machine learning methods, such as clustering algorithms, texture analysis techniques, and rule-based models, have demonstrated efficacy primarily due to their simplicity, computational efficiency, and interpretability. These methods typically involve handcrafted feature extraction strategies tailored to specific scar characteristics, enabling successful segmentation and classification tasks even with limited computational resources. However, the heavy reliance on manual feature engineering and the susceptibility of these methods to variations in image quality and environmental conditions restrict their generalization capability and applicability across diverse clinical scenarios.

CNN-based methods address many limitations inherent in traditional approaches by automatically extracting hierarchical features from extensive datasets, resulting in higher accuracy, improved robustness, and greater adaptability. CNN architectures such as ResNet, VGG, EfficientNet, and Mask R-CNN have demonstrated impressive results in tasks ranging from scar severity assessment and subtype classification to detailed collagen structure analysis. Despite their superior performance, CNN-based methods face challenges, including substantial computational demands, dependence on large-scale, high-quality annotated datasets, and concerns regarding interpretability and transparency, which remain critical barriers to widespread clinical adoption (160).

Hybrid approaches integrating 3D reconstruction techniques with deep learning represent a promising research frontier in scar assessment, offering precise quantitative analysis and non-contact measurement capabilities. These methods have shown exceptional promise for applications requiring accurate dimensional analysis, particularly in complex surface evaluations such as forensic investigations (112). However, the computational complexity, time-consuming data acquisition, and reliance on high-quality 3D data continue to pose significant practical challenges that need addressing through optimized computational strategies and improved imaging protocols.

Despite significant progress, several critical challenges persist in intelligent scar recognition research. A paramount limitation is the scarcity of standardized, publicly accessible scar image datasets, compounded by patient privacy and ethical considerations. The current dataset availability is insufficient to fully support robust model training and validation, impeding the reproducibility and generalization of research outcomes. Future advancements critically depend on establishing comprehensive, ethically sourced datasets, adopting standardized image acquisition protocols, and developing innovative data augmentation techniques to mitigate dataset limitations.

Additionally, future research should focus on addressing the interpretability and transparency of AI models to build trust among clinicians and ensure the practical applicability of these tools in real-world medical environments. Enhanced interpretability would allow clinicians not only to understand model predictions but also facilitate the integration of AI-based diagnostic tools within routine clinical workflows, ultimately improving patient care quality.

To address these challenges and propel the field forward, we propose several key research directions:

- **Development of hybrid models**: future research should focus on developing hybrid approaches that combine traditional image processing and deep learning techniques, such as linear-transformation-based dehazing to enhance scar image contrast and suppress illumination artifacts (161), and spectrum-based image enhancement methods that convert RGB images into narrowband or hyperspectral-like representations to boost dermatological lesion classification and detection performance (162–164). By leveraging the interpretability and low computational cost of classical preprocessing alongside the powerful feature extraction of modern networks, these hybrids can improve lesion boundary separability and enable more accurate, real-time scar diagnosis.
- **Improvement of dataset accessibility and quality**: the establishment of large-scale, publicly available datasets specifically curated for scar analysis is essential. Encouraging collaboration between clinical institutions, research communities, and regulatory bodies can facilitate the creation of diverse, ethically sourced datasets that adhere to strict privacy standards.
- **Clinical validation and trials**: rigorous clinical validation through well-designed prospective studies and multicenter clinical trials is crucial to evaluate the real-world effectiveness, reliability, and safety of intelligent diagnostic systems (165). Such validation efforts would ensure that AI technologies meet clinical standards and demonstrate tangible patient benefits.
- **Advancement in ethical AI application**: ethical considerations and patient privacy must be integral to the development and deployment of AI-based diagnostic tools. Implementing transparent, explainable AI practices and stringent data governance frameworks can address ethical concerns and foster broader acceptance among patients and healthcare providers (166).

In conclusion, the integration of intelligent diagnostic technologies into scar recognition and management signifies a transformative shift toward more objective, efficient, and patient-centered healthcare solutions. While substantial progress has been achieved, ongoing efforts in methodological innovation, dataset improvement, clinical validation, and ethical governance are essential for fully realizing the potential of AI in dermatological care. By addressing these critical aspects, future research will undoubtedly pave the way for the broader and more effective adoption of intelligent diagnostic systems, ultimately improving the quality of life for individuals affected by scars.
